# Mapping global trends in vaccine confidence and investigating barriers to vaccine uptake: a large-scale retrospective temporal modelling study

**DOI:** 10.1016/S0140-6736(20)31558-0

**Published:** 2020-09-26

**Authors:** Alexandre de Figueiredo, Clarissa Simas, Emilie Karafillakis, Pauline Paterson, Heidi J Larson

**Affiliations:** a The Vaccine Confidence Project, Department of Infectious Disease Epidemiology, London School of Hygiene & Tropical Medicine, London, UK; b Department of Mathematics, Imperial College London, London, UK; c Department of Health Metrics Sciences, University of Washington, Seattle, WA, USA; d Centre for the Evaluation of Vaccination, Vaccine & Infectious Disease Institute, University of Antwerp, Antwerp, Belgium

## Abstract

**Background:**

There is growing evidence of vaccine delays or refusals due to a lack of trust in the importance, safety, or effectiveness of vaccines, alongside persisting access issues. Although immunisation coverage is reported administratively across the world, no similarly robust monitoring system exists for vaccine confidence. In this study, vaccine confidence was mapped across 149 countries between 2015 and 2019.

**Methods:**

In this large-scale retrospective data-driven analysis, we examined global trends in vaccine confidence using data from 290 surveys done between September, 2015, and December, 2019, across 149 countries, and including 284 381 individuals. We used a Bayesian multinomial logit Gaussian process model to produce estimates of public perceptions towards the safety, importance, and effectiveness of vaccines. Associations between vaccine uptake and a large range of putative drivers of uptake, including vaccine confidence, socioeconomic status, and sources of trust, were determined using univariate Bayesian logistic regressions. Gibbs sampling was used for Bayesian model inference, with 95% Bayesian highest posterior density intervals used to capture uncertainty.

**Findings:**

Between November, 2015, and December, 2019, we estimate that confidence in the importance, safety, and effectiveness of vaccines fell in Afghanistan, Indonesia, Pakistan, the Philippines, and South Korea. We found significant increases in respondents strongly disagreeing that vaccines are safe between 2015 and 2019 in six countries: Afghanistan, Azerbaijan, Indonesia, Nigeria, Pakistan, and Serbia. We find signs that confidence has improved between 2018 and 2019 in some EU member states, including Finland, France, Ireland, and Italy, with recent losses detected in Poland. Confidence in the importance of vaccines (rather than in their safety or effectiveness) had the strongest univariate association with vaccine uptake compared with other determinants considered. When a link was found between individuals' religious beliefs and uptake, findings indicated that minority religious groups tended to have lower probabilities of uptake.

**Interpretation:**

To our knowledge, this is the largest study of global vaccine confidence to date, allowing for cross-country comparisons and changes over time. Our findings highlight the importance of regular monitoring to detect emerging trends to prompt interventions to build and sustain vaccine confidence.

**Funding:**

European Commission, Wellcome, and Engineering and Physical Sciences Research Council.

## Introduction

Alongside persisting issues of access to health services, waning vaccine confidence has taken a toll on immunisation programmes across the globe,^1^, ^2^, ^3^, ^4^ contributing to stagnating or decreasing immunisation rates and consequent surges in vaccine-preventable diseases such as measles.^5^, ^6^ In this context, WHO has named vaccine hesitancy as one of the top ten threats to global health in 2019 alongside climate change.^7^

Founded in 2010, the Vaccine Confidence Project (VCP) was established to develop systematic approaches to monitoring public confidence in vaccines and to inform policy makers and stakeholders of the changing trends and determinants of vaccine confidence across the globe. Over the past decade, the VCP has comprehensively explored the landscape of confidence issues and experiences in managing confidence crises around the world.^8^, ^9^, ^10^, ^11^ The VCP has conducted numerous surveys, focus groups, in-depth qualitative research, and large-scale digital media analytics,^12^, ^13^, ^14^ as well as convened expert roundtables and workshops to understand context-specific attitudes to vaccines among the general public,^14^, ^15^ health-care professionals and providers,^15^ and pregnant women.^16^ The VCP continues to research the roots, trends, and impacts of vaccine confidence issues at national and supranational levels to inform policy and trust-building activities and mitigate the need for crisis management in immunisation programmes.

Among a multiplicity of factors influencing vaccine decisions,^17^ key drivers of public confidence in vaccines were identified as trust in the importance, safety, and effectiveness of vaccines, along with compatibility of vaccination with religious beliefs.^18^ These findings have resulted in the development of a Vaccine Confidence Index (VCI) survey tool (first implemented in 2015^14^) to measure individual perceptions on the safety, importance, effectiveness, and religious compatibility of vaccines. The VCI questionnaire has the primary focus of measuring confidence across multiple countries while being minimal, thus allowing ready integration into existing global surveys. The VCI survey is one of a diverse set of metrics and indices used to measure confidence or hesitancy such as the Parent Attitudes About Childhood Vaccines Survey, which measures vaccine hesitancy among parents;^19^ the Vaccination Confidence Scale, which measures confidence in adolescent vaccination;^20^ the 5-C scale (confidence, complacency, constraints, calculation, and collective responsibility), which identifies psychological barriers of vaccination behaviour;^21^ and the SAGE Vaccine Hesitancy Scale, which has been deployed across multiple countries.^22^, ^23^, ^24^, ^25^, ^26^

**Research in context**

**Evidence before this study**

We have previously done three systematic reviews identifying the key determinants of vaccine hesitancy to inform questionnaire design around vaccine confidence. Vaccine refusals and delays are contributing to an increasing number of vaccine-preventable disease outbreaks (eg, measles and polio) globally. For this reason, vaccine hesitancy was named by WHO as one of the top ten threats to global health in 2019. Factors modulating vaccine confidence and, conversely, hesitancy include (but are not limited to) trust in health-care systems, providers, governance, information, and perceptions of vaccine importance, safety, and efficacy. The Vaccine Confidence Project (VCP) was founded 10 years ago to establish a systematic approach to monitoring public confidence in vaccines. In 2015, the VCP developed the Vaccine Confidence Index (VCI) survey: a tool used to monitor spatiotemporal trends in vaccine confidence at national and global levels.

**Added value of this study**

We used new and existing VCI surveys comprising nearly 300 000 individual responses from 149 countries around the world, allowing unprecedented insights into global time-varying trends in vaccine confidence. Datasets previously collected in collaboration with ORB International (Gallup International), the European Commission, and the Philippines Survey and Research Centre were used alongside novel data from the Sahel and worldwide data collected up to the end of 2019. We used Bayesian tools to estimate national-level vaccine confidence trends and to investigate the link in each country between vaccine uptake and socioeconomic and non-socioeconomic (eg, confidence in vaccine, trust in health system) determinants. To our knowledge, this is the largest study of global vaccine confidence to date, using a common metric to allow cross-country comparisons of vaccine sentiment globally.

**Implications of all the available evidence**

This study provides novel insights into worldwide variations in vaccine confidence and presents the country-dependent factors that modulate vaccine decisions. These include perceptions of vaccine safety, efficacy, and importance; socioeconomic and demographic determinants; and individually reported levels of trust. The study findings are discussed in light of past and ongoing vaccine confidence issues in different settings. A key implication of this analysis is the importance of regular monitoring of vaccine confidence levels to detect trends and changes that suggest the need for interventions to sustain confidence and pre-empt negative impacts on vaccination uptake.

In this large-scale retrospective study, we explore global trends in vaccine confidence between 2015 and 2019. We combine previously published data from nearly a quarter of a million individual survey responses with 50 000 additional interviews from 2019. To date, no comparable global estimates and monitoring of vaccine confidence are available, prohibiting a quantitative understanding of the relationship between vaccine coverage, socioeconomic demographics, and confidence. This analysis aims to provide multiyear global-level estimates of vaccine confidence for 149 countries worldwide, exploring trends in confidence and the global determinants of uptake including socioeconomic determinants and sources of trust.

## Methods

### Data sources

Between September, 2015, and December, 2019, reported vaccine confidence levels were collected from 284 381 individuals aged 18 years or older across 149 countries as part of 290 nationally representative surveys. One country was surveyed over six different timepoints (Philippines), 13 countries were surveyed over four timepoints, 28 over three timepoints, 40 over two timepoints, and 67 once. We grouped countries and territories by WHO regional classification. We classified the territories of Hong Kong, Northern Cyprus, and the occupied Palestinian territory into the Western Pacific, European, and Eastern Mediterranean regions, respectively.

Survey collection has been conducted through collaboration with ORB International (Gallup International), the European Commission,^15^ the Philippines Survey and Research Center,^27^ and Wellcome.^28^ Vaccine confidence was measured through three survey statements relating to individual perceptions on the importance, safety, and effectiveness of vaccines (table). Online, telephone, and face-to-face survey methodologies were used (see appendix 1 pp 3–4 for further details on survey methodologies). Responses to the three statements were answered on Likert scales ranging from “strongly disagree” to “strongly agree”. Likert responses presented to respondents differed between surveys (appendix 1 p 4); however, the outermost categories (“strongly agree” and “strongly disagree”) remained consistent across surveys. Thus, to allow meaningful comparisons in trends between surveys, individual survey responses falling between “strongly agree” and “strongly disagree” were recoded into the new category “neither strongly agree nor strongly disagree”, with the “strongly agree” and “strongly disagree” responses remaining unchanged. This reclassification assumes that respondents who report the most confident (“strongly agree”) and least confident (“strongly disagree”) beliefs are unlikely to change their response regardless of whether they are presented with additional Likert categories such as “tend to agree”, “somewhat agree”, or “neither agree nor disagree” (or a combination thereof; appendix 1 p 4).

**Table:** Data used throughout the study

	**Responses**	**Baseline for univariate Bayesian regressions**
Vaccine confidence*		
“I think vaccines are safe”	Likert scale recoded to “strongly disagree”, “strongly agree”, and “neither strongly agree nor strongly disagree”	Not strongly agree
“I think vaccines are important for children to have”	As above	As above
“I think vaccines are effective”	As above	As above
**Vaccine uptake**†		
If respondent has children: “…have any of your children ever received a vaccine that was supposed to prevent them from getting childhood diseases…?”	Yes, no, do not know; “do not know” responses are recoded to “no”	No
**Source of trust**		
“[Who] do you trust most to give you medical or health advice?”	Family and friends (social circle), a doctor or nurse, other sources (famous people, traditional healers, or none)	A doctor or nurse
“[How much] do you trust medical and health advice from the government…?”	A lot, some, not much, not at all; responses are recoded to “high” (a lot) and “low” (others)	Low
“[How much] do you trust medical and health advice from medical workers, such as doctors and nurses…?”	A lot, some, not much, not at all; responses are recoded to “high” (a lot) and “low” (others)	Low
“How much do you trust…traditional healers…?”	A lot, some, not much, not at all; responses are recoded to “high” (a lot) and “low” (others)	Low
**Information seeking**		
“Have you…tried to get any information about medicine, disease, or health in the past 30 days?” and “Would you…like to know more about medicine, disease, or health?”	A joint information-seeking behaviour variable is defined with responses “high” (if “yes” answered to both statements) and “low” (otherwise)	Low
**Demographics and socioeconomic status**		
Sex	Male or female	Female
Age	Integer-valued age, scaled to have a mean of 0 and unit SD	No baseline
Income quintile	Quintiles: Q1 (lowest income) to Q5 (highest income)	Q1
Religion: “Could you tell me what your religion is?”	For each country, religion is recoded into the most frequently reported religion in a given country (largest), all other religious affiliations (minority), or refusal to answer (refused)	Other
Education	Years in education grouped into <9 years, 9–15 years, and ≥16 years	<9 years
Science education: “Have you…learned about science at [school]?”	Primary, secondary, university, no, do not know; “primary” and “do not know” responses are recoded to “low”, with all others to “high”	Low

* As data on perceptions about the religious compatibility of vaccines was not posed to respondents in the Wellcome Global Monitor, we only consider three statements on vaccine confidence.

† For this question, vaccine-preventable diseases were given as examples to respondents, which varied by country (see appendix 1 pp 28–29).

Of the 290 surveys, 144 were collected as part of the 2018 Wellcome Global Monitor (WGM; appendix 2).^28^ In addition to probing individuals' perceptions on vaccine confidence across the globe, the WGM also surveys individuals on a range of factors including sources of trust, information-seeking behaviours, and whether respondents with children report having vaccinated at least one child against any routine immunisation programme (if the respondent reports having at least one child). To explore barriers to vaccine uptake, we extracted data from the WGM surveys on demographics (sex, age, and religious beliefs) and socioeconomic status (income and education, including science education), in addition to sources of trust and information-seeking behaviours. These variables are summarised in the table.

Missing data by country are listed in appendix 1 (pp 25–26). On average, each survey contained approximately 1000 individuals. The surveys were weighted by sex and age according to national distributions, with equal sex representation in most surveys (appendix 2).

### Model-based estimates of vaccine confidence

The proportion of respondents falling into each of the three response categories (“strongly agree”, “strongly disagree”, and “neither strongly agree nor strongly disagree”) for each confidence statement and in each country at any given timepoint was modelled as multinomial logit Gaussian process model.^29^ Model inference was done using Gibbs sampling and 10 000 samples (or draws) were obtained from the posterior predictive distributions, from which mean estimates were calculated (see appendix 1 pp 5–7 for full model details). Model performance was assessed using out-of-sample validation using five-fold cross-validation. Out-of-sample metrics indicated good model fit with a mean error of 10^−17^, mean absolute error of 10·10, and a root mean square error of 15·29. Our model was used to estimate vaccine confidence across all surveyed countries at two timepoints (November, 2015, and November, 2018) when many surveys were conducted. To evaluate more recent temporal trends, changes in confidence estimates under our model are provided between November, 2015, and December, 2019. More recent changes in vaccine safety perceptions are evaluated for the EU, where a higher frequency of surveys has been conducted, on average, compared with the rest of the world. A difference of posterior distributions of the probability of strongly agreeing (and strongly disagreeing) is calculated between each country's two most recent datapoints from 2018 onwards spaced at least 12 months apart (see appendix 2 for further details). A change in confidence is recorded if the 95% highest posterior density (HPD) interval of this difference distribution excludes zero. The same methodology is used to identify countries worldwide with an increase in the proportions of respondents strongly disagreeing that vaccines are safe, important, or effective between November, 2015 and December, 2019.

### Vaccine uptake determinants

Bayesian logistic regressions were used to investigate the link in each country between vaccine uptake and confidence, source of trust, information-seeking behaviour, and demographics and socioeconomic status (table).^30^ This analysis serves to highlight consistent trends in uptake determinants across the globe and provides a platform for deeper analysis examining the most important factors of uptake in each country or developing predictive models of vaccine uptake.

For the vaccine uptake analysis, we included respondents from the WGM dataset who reported having heard of vaccinations and having had children. The strength of the relationship between the percentage of respondents in each country strongly agreeing that vaccines are important, safe, and effective and the percentage of respondents reporting having had their children vaccinated was assessed using Pearson's correlation coefficient, where percentages *p* were first transformed onto the real line using −log(100/*p* – 1).

All models were implemented in JAGS^31^ using R (version 3.6.1). 95% HPD intervals were used to represent confidence in parameter estimates. The 95% HPD interval is the smallest interval of the posterior distribution that contains 95% of the probability mass. Raw survey data for all 290 surveys can be found in appendix 2, alongside model-based vaccine confidence estimates from November, 2015, to January, 2020.

### Role of the funding source

The funders of the study had no role in study design, data collection, data analysis, data interpretation, or writing of the report. The corresponding author had full access to all the data in the study and had final responsibility for the decision to submit for publication.

## Results

**Figure 1:**
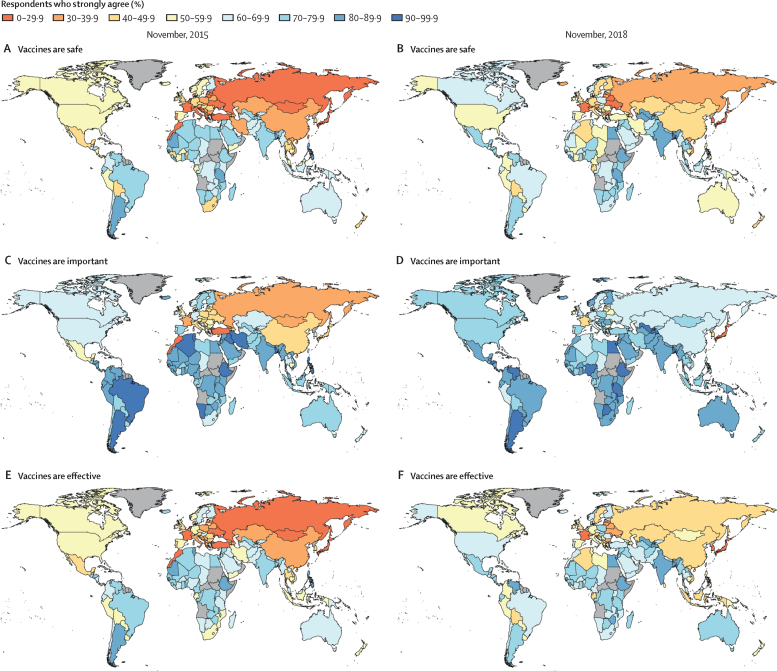
Global trends in perceptions towards the safety of vaccines in November, 2015, and November, 2018 Figure shows model-based estimates of the percentage of respondents strongly agreeing that vaccines are safe (panels A, B), important for children to have (panels C, D), and effective (panels E, F) in November, 2015, and November, 2018. No data were available for countries in grey.

Model-based estimates of the percentage of respondents strongly agreeing that vaccines are safe, important, and effective in 2015 and 2018 are shown in figure 1. Estimates for all countries with associated uncertainties are shown in the appendices, alongside time-series plots showing temporal trends in vaccine confidence (appendix 1 pp 8–23; appendix 2).

Argentina (89·4%, 95% HPD interval 87·7–91·3), Liberia (86·1%, 67·1–97·7), and Bangladesh (86·1%, 83·7–88·1) had the highest estimated percentage of respondents strongly agreeing that vaccines are safe in late 2015, whereas Japan (8·9%, 7·4–10·6), France (8·9%, 7·2–10·5), and Mongolia (8·1%, 6·4–9·8) had the lowest (figure 1A).

Ethiopia (96·3%, 95% HPD interval 95·2–97·3), Argentina (95·7%, 94·5–97·0), and Bangladesh (95·1%, 93·8–96·4) had the highest estimated percentage of respondents strongly agreeing that vaccines are important in 2015, whereas Turkey (22·1%, 19·5–24·7), Morocco (15·8%, 13·7–18·4), and Georgia (2·7%, 1·6–3·8) had the lowest (figure 1C).

Ethiopia (86·6%, 84·5–88·7), Argentina (86·3%, 84·3–88·4), and Mauritania (81·9%, 64·4–97·2) had the highest estimated percentage of respondents strongly agreeing that vaccines are effective in late 2015, whereas Japan (14·7%, 12·5–16·7), Mongolia (13·0%, 11·0–15·2), and Morocco (10·3%, 8·6–12·2) had the lowest (figure 1E).

**Figure 2:**
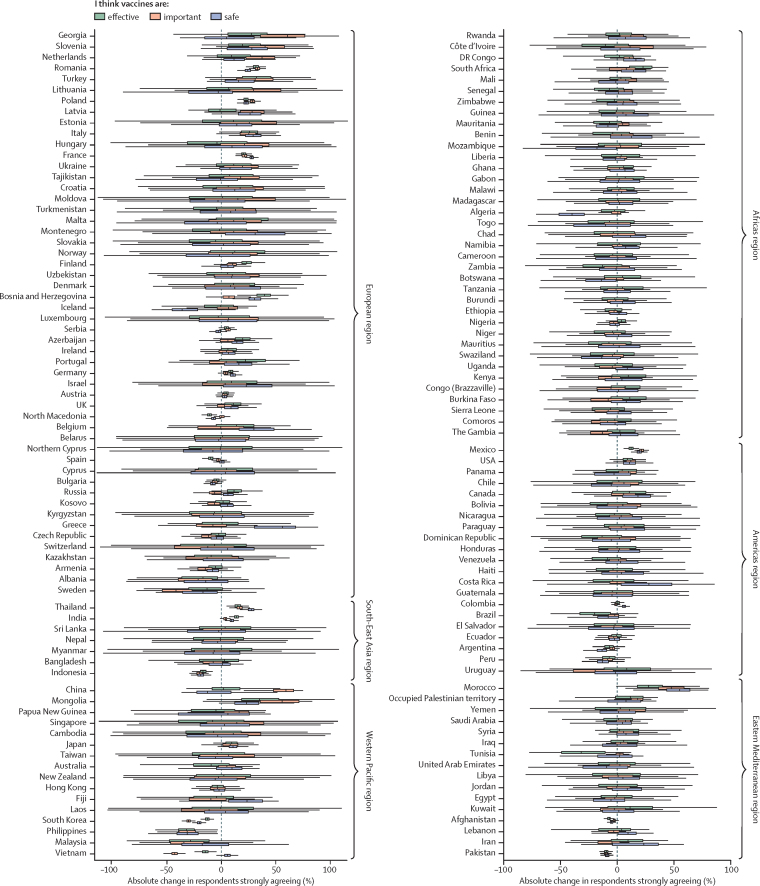
Distributions in absolute confidence changes between November, 2015, and December, 2019 Distributions of model-based estimates in the absolute differences in the proportions of respondents strongly agreeing that vaccines are safe, important, and effective. Positive values denote an increase in confidence between 2015 and 2019. Owing to increased uncertainty around estimates further away from survey dates, some significant changes in confidence over the study period are not captured by this figure.

Between November, 2015, and December, 2019, we estimate that vaccine confidence fell for all three elements of confidence in Indonesia, the Philippines, Pakistan, and South Korea, and for two elements in Afghanistan and Vietnam (figure 2). The Philippines, which ranked in the top ten countries worldwide in late 2015 for vaccine confidence for all three elements, ranked no higher than 70th in 2019, having experienced a large fall in the percentage of respondents strongly agreeing that vaccines are safe (absolute difference 23·1%, 95% HPD interval 6·3–40·0), important (23·0%, 7·4–37·4), and effective (23·7%, 8·5– 39·7; figure 2). Indonesia also witnessed large drops in confidence over this time in all three elements: vaccine safety (absolute difference 13·8%, 9·0–18·9), importance (14·6%, 9·9–19·5), and effectiveness (12·2%, 7·5–17·7; figure 2). Vaccine confidence increased between 2015 and 2019 across all three elements for France, India, Mexico, Poland, Romania, and Thailand (figure 2).

**Figure 3:**
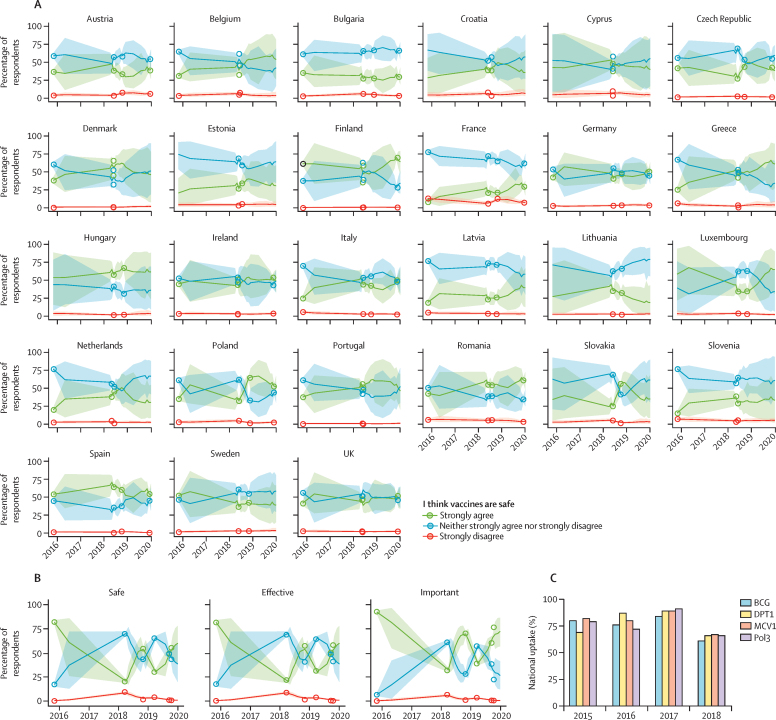
Trends in the perceived safety of vaccines in the EU and the Philippines (A) Time series of estimated percentages of respondents in EU countries strongly agreeing, strongly disagreeing, or neither strongly agreeing nor strongly disagreeing that vaccines are safe. Lines are means and shaded regions are 95% HPD intervals. Circles show the observed percentage of respondents from raw data (appendix 2). Time series for all countries for all three confidence statements are shown in appendix 1 (pp 12–23). (B) Time series of survey responses across all three survey questions for the Philippines. (C) WHO-UNICEF national immunisation estimates for routine vaccination programmes in the Philippines against tuberculosis (BCG), diphtheria-pertussis-tetanus (DPT1), measles (MCV1), and polio (Pol3). HPD=highest posterior density.

Time-series trends in vaccine confidence until the end of 2019 are shown for the EU (including the UK) and the Philippines (figure 3). Recent losses in the percentage of respondents agreeing that vaccines are safe (between 2018 and late 2019) are detected in Poland, with increases detected in Finland, France, Ireland, Italy, and the UK (figure 3A; appendix 2). Vaccine confidence plummeted between 2015 to 2018 in the Philippines (over well documented fears around the Dengvaxia vaccine in 2017^27^, ^32^, ^33^); however, since the start of 2018, confidence in the importance of vaccines has made substantial gains, with less substantial increases in vaccine safety and effectiveness perceptions (figure 3B). The loss of vaccine confidence in the Philippines triggered by fears over Dengvaxia appears to also have affected uptake of routine vaccines recommended by the national immunisation programme (figure 3C).^27^ This pattern is not limited to the Philippines: there were larger increases in the percentage of respondents perceiving vaccines to be important than safe or effective across the majority of countries in the European region for which there was an improvement in recorded importance confidence (figure 2).

For ten countries worldwide, our model estimated a higher percentage of respondents strongly disagreeing that vaccines are safe, important, or effective in December, 2019, compared with November, 2015 (appendix 1 p 24): Afghanistan, Azerbaijan, Bosnia and Herzegovina, Georgia, Indonesia, Japan, Malaysia, Nigeria, Pakistan, and Serbia. Of note, Afghanistan, Azerbaijan, Indonesia, Nigeria, Pakistan, and Serbia all had increased concerns about vaccine safety (appendix 1 p 24)

**Figure 4:**
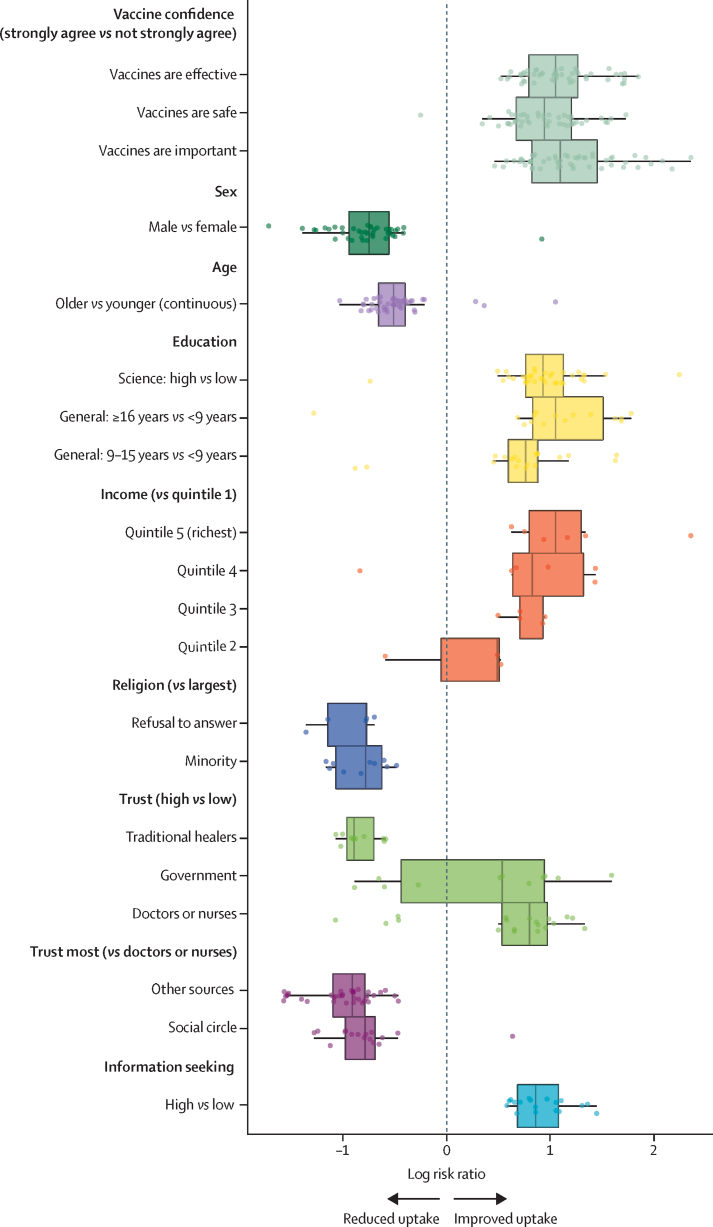
Univariate determinants of vaccine uptake within the Wellcome Global Monitor dataset Global trends in univariate associations between vaccine uptake and confidence in vaccines, demographics and socioeconomic status, sources of trust, and information-seeking behaviours. Each point represents a significant association (95% HPD interval excludes zero) between a variable and uptake in a given country. Boxplots show the median log risk ratio and IQR. All variables (except age, which is continuous) are categorical and baseline groups are specified by each category (eg, high *vs* low denotes low as the baseline group; see the table for definitions). HPD=highest posterior density.

Significant associations between uptake and vaccine confidence, sources of trust, information-seeking behaviour, and demographics and socioeconomic status are shown in figure 4, with consistent trends shown across the globe. Overall, the determinants most consistently associated with improved uptake were high confidence in vaccines (66 countries); trusting health-care workers more than family, friends, or other non-medical sources for medical and health advice (43 countries); higher levels of science education (35 countries); sex, with women more likely than men to report any child having at least one vaccine in 41 countries and men more likely than women in just one country (Chad); age (younger age groups associated with increased chances of uptake in 43 countries); and high information-seeking behaviour (18 countries). Income and religion were less widely associated with uptake; however, when a link was found between religion and uptake, it is the minority religious groups (or those refusing to provide their religious belief) who were associated with lower probability of uptake (figure 4).

**Figure 5:**
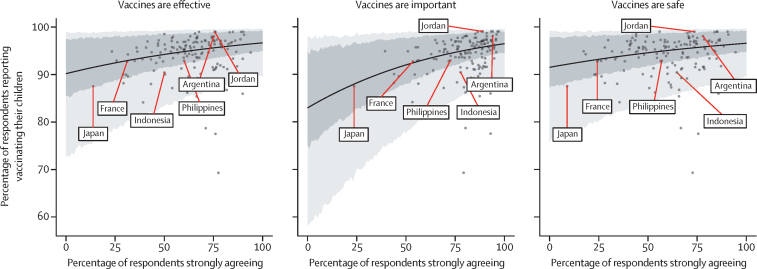
Association between national level vaccine uptake and vaccine confidence as reported in the Wellcome Global Monitor The solid line represents the mean regression with 70% and 95% HPD intervals shaded in dark and light grey, respectively. Datapoints for (lowest confidence) and the Philippines (case study) are shown, alongside randomly selected countries. HPD=highest posterior density.

Countries with higher percentages of respondents strongly agreeing that vaccines are safe, important, and effective had higher percentages of respondents reporting that they have had their children vaccinated (WGM data only; figure 5). These effect sizes are small but significant, with a Pearson's correlation of 0·28 (95% CI 0·12–0·42) between the percentage strongly agreeing that vaccines are safe and the percentage of respondents reporting vaccinating their children across all countries, 0·45 (0·31–0·57) for vaccine importance and uptake, and 0·28 (0·12–0·42) for vaccine effectiveness and uptake.

## Discussion

To our knowledge, this is the largest study of vaccine confidence, offering important insights into the global state of vaccine confidence. Vaccine confidence was estimated and mapped for 149 countries from 2015 to 2019, revealing key spatial and temporal trends. Although confidence remained low across Europe compared with other continents, there are signs that vaccine confidence is increasing for much of Europe, including France, where vaccine confidence has been persistently low since 2015. Confidence increased overall in Poland between 2015 and 2019, but a recent loss in confidence was seen between late 2018 and late 2019, reflecting the growing impact of a highly organised local anti-vaccine movement.^34^

Between 2015 and 2018, vaccine confidence plummeted in the Philippines and Indonesia. In 2017, the vaccine manufacturer Sanofi announced that their newly introduced dengue vaccine Dengvaxia posed a risk to individuals who had not previously been exposed to the virus, prompting outrage and panic across the population where nearly 850 000 children had been given the new vaccine the previous year. As the VCP measured a baseline confidence value in 2015, we were able to measure the change in confidence following the vaccine scare and found a significant drop in confidence in vaccine importance, safety, effectiveness.^27^ The VCI survey tool has detected a rise in confidence across the country—although confidence is not back to 2015 levels—indicating a possible recovery and highlighting the value of the tool in assessing the effectiveness of national-level policy.

Japan ranked among the countries with the lowest vaccine confidence in the world: this might be linked to the human papillomavirus (HPV) vaccine safety scares that started in 2013, and following the decision by the Japanese Ministry of Health, Labour and Welfare in June, 2013, to suspend proactive recommendation of the HPV vaccine.^35^ As a result of this vaccine safety scare, HPV vaccination coverage decreased from 68·4–74·0% in the 1994–98 birth cohort to 0·6% in the 2000 birth cohort.^36^ The news of Japan suspending their proactive recommendation of the HPV vaccine has travelled globally through online media and social media networks, being applauded by anti-vaccination groups but not by the global scientific community.^4^ The way in which the HPV vaccine scare was approached by health officials, as well as an ongoing outbreak of rubella in Japan,^37^, ^38^ indicate continuing issues with the Japanese vaccination programme that need resolving.^39^

Indonesia witnessed a large drop in confidence between 2015 and 2019, partly triggered by Muslim leaders questioning the safety of the measles, mumps, and rubella (MMR) vaccine, and ultimately issuing a *fatwa*—a religious ruling—claiming that the vaccine was *haram* and contained ingredients derived from pigs and thus not acceptable for Muslims. Local healers promoting natural alternatives to vaccines also contributed to the waning confidence in vaccines.^40^, ^41^

Results from our survey can inform the need for further research, to explore why certain countries might experience sudden increases or decreases in confidence. We have highlighted countries with marked decreases in percentages reporting that they strongly agree that vaccines are safe and countries with significant increases in those strongly disagreeing that vaccines are safe. These countries are candidates for more nuanced follow-up surveys to understand the precise drivers of confidence and the link between confidence and uptake.

In South Korea and Malaysia, online mobilisation against vaccines has been identified as a key barrier to vaccination.^42^, ^43^ The internet is a main source of vaccination information in Malaysia, where misinformation has been identified as influencing vaccine reluctance.^44^ In South Korea, an online community named ANAKI (Korean abbreviation of “raising children without medication”) has been strongly advocating against childhood immunisation.^45^ Future studies in both countries should further investigate this trend and propose mitigation strategies. In Georgia, unfounded vaccine safety concerns, amplified by the media, were found to profoundly affect a nationwide MMR vaccine campaign in 2008.^46^ Our findings of low vaccine confidence in Georgia could suggest that concerns about vaccine safety are again on the rise.

The determinants of vaccine uptake across the globe show strong consistency, with being male or having fewer years of education associated with decreased chances of uptake. Positive information-seeking behaviours and trusting health-care workers more than other sources such as one's social circle for medical and health advice were associated with increased chances of uptake.

There are several study limitations to note. First, as not all surveys used have consistent responses, we have made a key assumption that, presented with different options between the extreme categories of “strongly agree” and “strongly disagree” (which are consistent across all surveys), respondents with the strongest sentiment will fall into one of these extreme groups regardless of additional categories. While this approach probably allows meaningful comparison across surveys—although it needs testing for validation—it pools vaccination beliefs among those without the strongest beliefs, masking potentially key information. Second, the WGM survey data only permit an investigation of uptake defined as whether a parent has had any of their children vaccinated against at least one childhood disease. These uptake data are therefore not defined on a vaccine or child-by-child basis, precluding an investigation of determinants of vaccine-specific or child-specific uptake (which has been done recently in the literature^22^, ^24^, ^41^). Moreover, we rely on parental recall being accurate, and patterns of recall error not varying substantially across countries. Finally, owing to low case counts of respondents who have not had their children vaccinated (appendix 2) and the varying religious groups across countries, religious groups were recoded into the largest and minority groups to extract results from our regression analysis. In many settings, more nuanced regression findings are possible, and a comprehensive regression analysis could reveal more informative country-specific determinants of vaccine uptake.

Sentiments seeding doubt and distrust and the viral spread of misinformation are contributing to a landscape of uncertainty. Some actors have purposefully polarised vaccine debates, exploiting the doubting public and system weaknesses for political purposes,^47^, ^48^ while waning vaccine confidence in other settings might be influenced by a wider environment of distrust in government and scientific elites.^14^ The findings of declining confidence in Afghanistan, Azerbaijan, Pakistan, and Nigeria mirror trends in political instability and religious extremism in these settings.^49^ Over the past few years in Pakistan and Nigeria, new waves of misinformation surrounding the polio vaccine have been circulating,^50^, ^51^ and have led to recent increases in poliovirus cases in both countries.^52^ Further research should investigate the link between political polarisation, religious extremism, and populism and vaccination beliefs to better understand these complex ties.^48^ Having a common metric of confidence and a baseline for comparison is crucial to understanding these changing trends over time, which can serve as an early warning system to prompt needed intervention to avert drops in vaccine confidence and acceptance.

In the context of new and emerging disease outbreaks, such as the COVID-19 pandemic, the VCI provides a valuable baseline of confidence levels to measure change in times of evolving disease threats and to help to identify where more trust building is needed to optimise uptake of new life-saving vaccines.

## Data sharing

All raw data used as input to our multinomial Gaussian process model (appendix 1 pp 5–6) can be found in appendix 2.
