# Supplementary material for: Mapping global trends in vaccine confidence and investigating barriers to vaccine uptake: a large-scale retrospective temporal modelling study
Source: Lancet. 2020 Sep 26;396(10255):898–908. doi: 10.1016/S0140-6736(20)31558-0 (PMC7607345; doi:10.1016/S0140-6736(20)31558-0)
Supplement: Supplementary appendix 1 [file mmc1.pdf]

THE LANCET

Supplementary appendix

This appendix formed part of the original submission and has been peer reviewed. We post it as supplied by the authors.

Supplement to: de Figueiredo A, Simas C, Karafillakis E, Paterson P, Larson HJ. Mapping global trends in vaccine confidence and investigating barriers to vaccine uptake: a large-scale retrospective temporal modelling study. *Lancet* 2020; published online Sept 10. http://dx.doi.org/10.1016/S0140-6736(20)31558-0.

Supplementary Materials: Mapping global trends in vaccine confidence and investigating barriers to vaccine uptake: a large-scale retrospective temporal modelling study

Alexandre de Figueiredo, PhD; Clarissa Simas, MSc; Emilie Karafillakis, MSc; Pauline Paterson, PhD; and Heidi J. Larson, PhD.

Contents

1 Data **3**

1.1 Survey information and fieldwork dates . . . 3

2 Estimating vaccine confidence **5**

2.1 Model . . . 5

2.2 Prior distributions . . . 6

2.3 Model performance . . . 7

3 Model-based estimates of confidence and associated uncertainty intervals **8**

4 Global trends in vaccine confidence **12**

4.1 I think vaccines are safe . . . 12

4.2 I think vaccines are important . . . 16

4.3 I think vaccines are effective . . . 20

4.4 Decreases in vaccine confidence . . . 24

5 Vaccine uptake and its socioeconomic determinants **25**

5.1 Data summaries . . . 25

5.2 Missing data . . . 25

5.3 A note on Bayesian logistic regression . . . 27

6 Vaccines in Wellcome Global Monitor **28**

1 Data

1.1 Survey information and fieldwork dates

The Vaccine Confidence Index^{TM}(VCI^{TM}) is a four item questionnaire that measures individuals' perceptions on the importance, safety, effectiveness, and religious compatibility of vaccines via the four statements: overall I think vaccines are important for children to have; overall I think vaccines are safe; overall I think vaccines are effective; and vaccines are compatible with my religious beliefs.

These four items[1] have been included in multiple surveys between 2015 and 2019 (Table 1). Survey methodology differs by survey, with face-to-face, telephone, and online surveys used (see references in Table 1). Respondents aged 18 and over were considered eligible for our study. Responses are collected on a Likert scale ranging from "strongly disagree" to "strongly agree". However, interim categories differ between surveys (Table 1). As a result, the internal categories (i.e. not "strongly agree" or "strongly disagree") are reclassified into "neither agree nor disagree". The assumption in this reclassification is that those who report the most extreme responses on these scales will do so regardless of an additional "neither agree nor disagree" category nor a change of wording from 'somewhat' to 'tend to' in the adjacent categories.

Surveys are nationally representative with respect to age, sex, and sub-national region.

[1]with the exception of Q4 which was not asked in the Wellcome Global Monitor.

Survey	C	N	Fieldwork dates	Q1-4 responses	Further details
WIN/Gallup International Association [1]	67	66,561	September to December 2015	strongly agree, tend to agree, tend to disagree, strongly disagree, do not know	Vaccine Confidence Index questionnaire included with WIN/Gallup International Association's End of Year Survey 2015 [1]. Data previously published by the authors in [2]. For the purposes of time-series estimation of confidence, all survey dates set to 31 October 2015.
WIN/Gallup International Association	6	12,761	November to December 2016	strongly agree, tend to agree, tend to disagree, strongly disagree, do not know	-
State of Vaccine Confidence in the EU (European Commission) [3]	28	28,780	May 2018 to June 2018	strongly agree, tend to agree, tend to disagree, strongly disagree, do not know	See Table 7, page 52 in [3] for exact survey dates.
Wellcome Global Monitor 2018 [4]	144	131,562	April 2018 to January 2019	strongly agree, somewhat agree, neither agree nor disagree, somewhat disagree, strongly disagree, do not know	Vaccine Confidence Index questionnaire included in the Wellcome Global Monitor 2018. See Wellcome Global Monitor 2018 Methodology [5] for exact survey dates.
Philippine Survey and Research Center [6]	1	1,500; 1,800; and 1,800	March 2018; December 2018; March 2019	strongly agree, somewhat agree, somewhat disagree, strongly disagree, do not know	Vaccine Confidence Index questionnaire conducted in three surveys in the Philippines.
WIN/Gallup International Association [7]	41	39,617	October 2019 to December 2019	strongly agree, tend to agree, tend to disagree, strongly disagree, do not know	Vaccine Confidence Index questionnaire included with WIN/Gallup International Association's End of Year Survey 2019.

Table 1: Survey methodologies, number of countries surveyed in each survey (C), sample sizes (N), fieldwork dates, survey Likert responses to vaccine confidence surveys, and further details

2 Estimating vaccine confidence

2.1 Model

The proportion of respondents falling into each of the three response categories (strongly agree, strongly disagree, and neither) in each country at each time is modelled as a multinomial logit Gaussian process (GP) model (see, e.g., [8, 9, 10]). For each country c and survey time t, we have observed count data $\mathbf{y}_{ct} = (y_{ct}^{\mathrm{SA}}, y_{ct}^{\mathrm{N}}, y_{ct}^{\mathrm{SD}})$, where each element is the (weighted) number of survey respondents who strongly agree (SA), strongly disagree (SD), or neither strongly agree nor strongly disagree (N) that vaccines are safe (or important or effective). We model the class membership probability as a latent Gaussian process

$$\boldsymbol{y}_{ct} \sim \mathrm{Multinomial}\big(\boldsymbol{p}_{ct}, n_{ct}\big), \tag{1}$$

where $\boldsymbol{p}_{ct} = (p_{ct}^{\mathrm{SA}}, p_{ct}^{\mathrm{N}}, p_{ct}^{\mathrm{SD}})$ are the class membership probabilities to be inferred and n_{ct} are the survey sample sizes. Gaussian process priors are placed over the log of the ratios $\frac{p^{\mathrm{SA}}}{p^{\mathrm{SD}}}$ and $\frac{p^{\mathrm{N}}}{p^{\mathrm{SD}}}$. Letting $\beta_{ct}^1 = \log \frac{p^{\mathrm{SA}}}{p^{\mathrm{SD}}}$ and $\beta_{ct}^2 = \log \frac{p^{\mathrm{N}}}{p^{\mathrm{SD}}}$, then:

$$\beta_{ct}^1 = \alpha_{cj}^1 + \epsilon_{ct}^1 + \delta_{jt}^1 \tag{2}$$

$$\beta_{ct}^2 = \alpha_{cj}^2 + \epsilon_{ct}^2 + \delta_{jt}^2, \tag{3}$$

where j denotes the region to which country c is classified according to the WHO[2] and where $\boldsymbol{\epsilon}_c^1$ and $\boldsymbol{\epsilon}_c^2$ are mean-zero GPs that account for country-level temporal correlation, while $\boldsymbol{\delta}_c^1$ and $\boldsymbol{\delta}_c^2$ are mean-zero GPs accounting for regional trends. These GPs are specified as,

$$\boldsymbol{\epsilon}^1 \sim \mathcal{N}\big(\mathbf{0}, \Sigma_{l_1^\epsilon, \sigma_1^\epsilon}\big) \tag{4}$$

$$\boldsymbol{\epsilon}^2 \sim \mathcal{N}\big(\mathbf{0}, \Sigma_{l_2^\epsilon, \sigma_2^\epsilon}\big) \tag{5}$$

$$\boldsymbol{\delta}^1 \sim \mathcal{N}\big(\mathbf{0}, \Sigma_{l_1^\delta, \sigma_2^\delta}\big) \tag{6}$$

$$\boldsymbol{\delta}^2 \sim \mathcal{N}\big(\mathbf{0}, \Sigma_{l_2^\delta, \sigma_2^\delta}\big), \tag{7}$$

[2] See `https://www.who.int/choice/demography/mortality_strata/en/`

where $\Sigma_{l,\sigma}$ are squared-exponential covariance functions parameterised by a characteristic length scale (l) and a signal variance (σ^2). Specifically, these covariance functions are given as,

$$\Sigma_{l,\sigma}(t_i, t_j) = \sigma^2 \exp\left(- \frac{(t_i - t_j)^2}{2l^2} \right), \tag{8}$$

when $t_i \neq t_j$ and $\sigma^2 + \sigma_n^2$ otherwise, where σ_n^2 is signal noise.

2.2 Prior distributions

Before fitting the model survey times are scaled to be on the interval [0, 1]. For each signal noise parameter $\sigma \sim \text{Gamma}(1, 1)$. Country-specific length-scales are given the priors,

$$l^{\epsilon} \sim \text{Unif}(0.1, 1). \tag{9}$$

As survey data was collected over a total of 50 months, this prior distribution restricts the minimum timescale to be 5 months, which we believe to loosely correspond to a minimum time-scale over which a full-blown confidence crises could develop (such as recently in the Philippines [11]). Region-specific length-scales are given a much longer characteristic time-scale, as one would expect continental trends to develop over longer periods of time,

$$l^{\delta} \sim \text{Unif}(1, 10). \tag{10}$$

Hierarchical priors are placed over the parameters α_{cj} to partially pool estimates towards regional means,

$$\alpha^1_{cj} \sim \mathcal{N}(a_{1j}, s^2_{a_1}) \tag{11}$$

$$\alpha^2_{cj} \sim \mathcal{N}(a_{2j}, s^2_{a_2}), \tag{12}$$

where $\tau_{a_1} = 1/s^2_{a_1} \sim \text{Gamma}(1, 1)$ and $\tau_{a_2} = 1/s^2_{a_2} \sim \text{Gamma}(1, 1)$, and $a_{1j} \sim \mathcal{N}(0, 10)$ and $a_{2j} \sim \mathcal{N}(0, 10)$. These prior choices over the country-level parameters α are reasonably uninformative priors over the plausible ranges of values for α.s

In all models 5,000 burn in steps and 10,000 sampling iterations are used after successful adaptation. Convergence and burn in are verified using the Geweke statistic [12].

2.3 Model performance

Model performance was assessed using five-fold cross validation. Out-of-sample metrics indicated good model fit with a mean error of 10^{-17} (indicating no upwards or downwards prediction bias), mean absolute error of 10.10, an RMSE of 15.29, and an out-of-sample Pearson correlation coefficient between observed and predicted values of 0.84.

3 Model-based estimates of confidence and associated uncertainty intervals

Figures 1-3 below extends main-text Figure 1 to show associated uncertainty intervals.

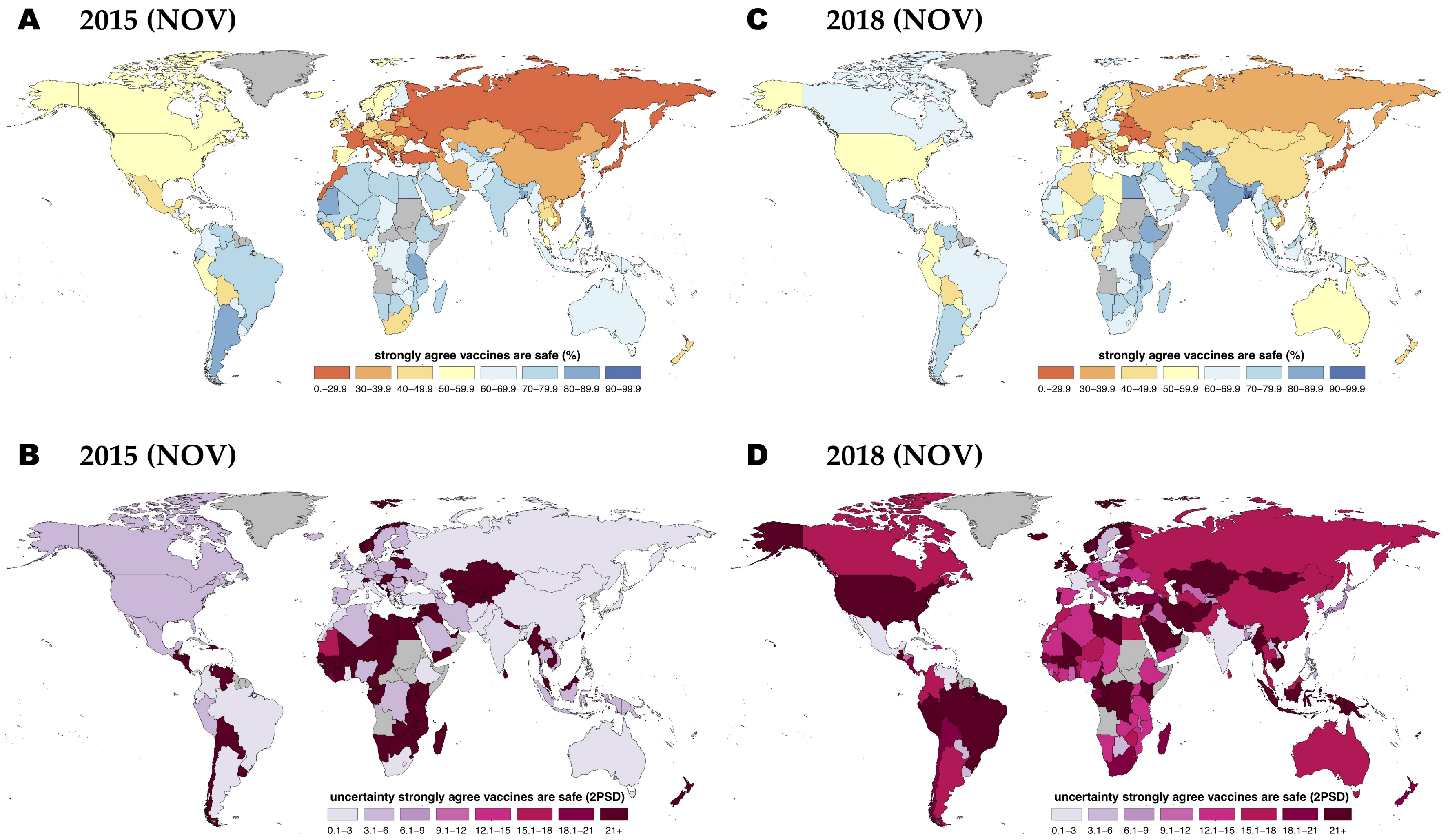

Figure 1: **A** Model-based estimates of the percentage of respondents strongly agreeing that vaccines are safe in November 2015 **B** Uncertainty in the estimate of the percentage of respondents strongly agreeing that vaccines are safe in November 2015 (two standard deviations of the posterior predictive distribution [2PSD]) **C** Model-based estimates of the percentage of respondents strongly agreeing that vaccines are safe in November 2018 **D** Uncertainty in the estimate of the percentage of respondents strongly agreeing that vaccines are safe in November 2019. No data were available for countries in grey.

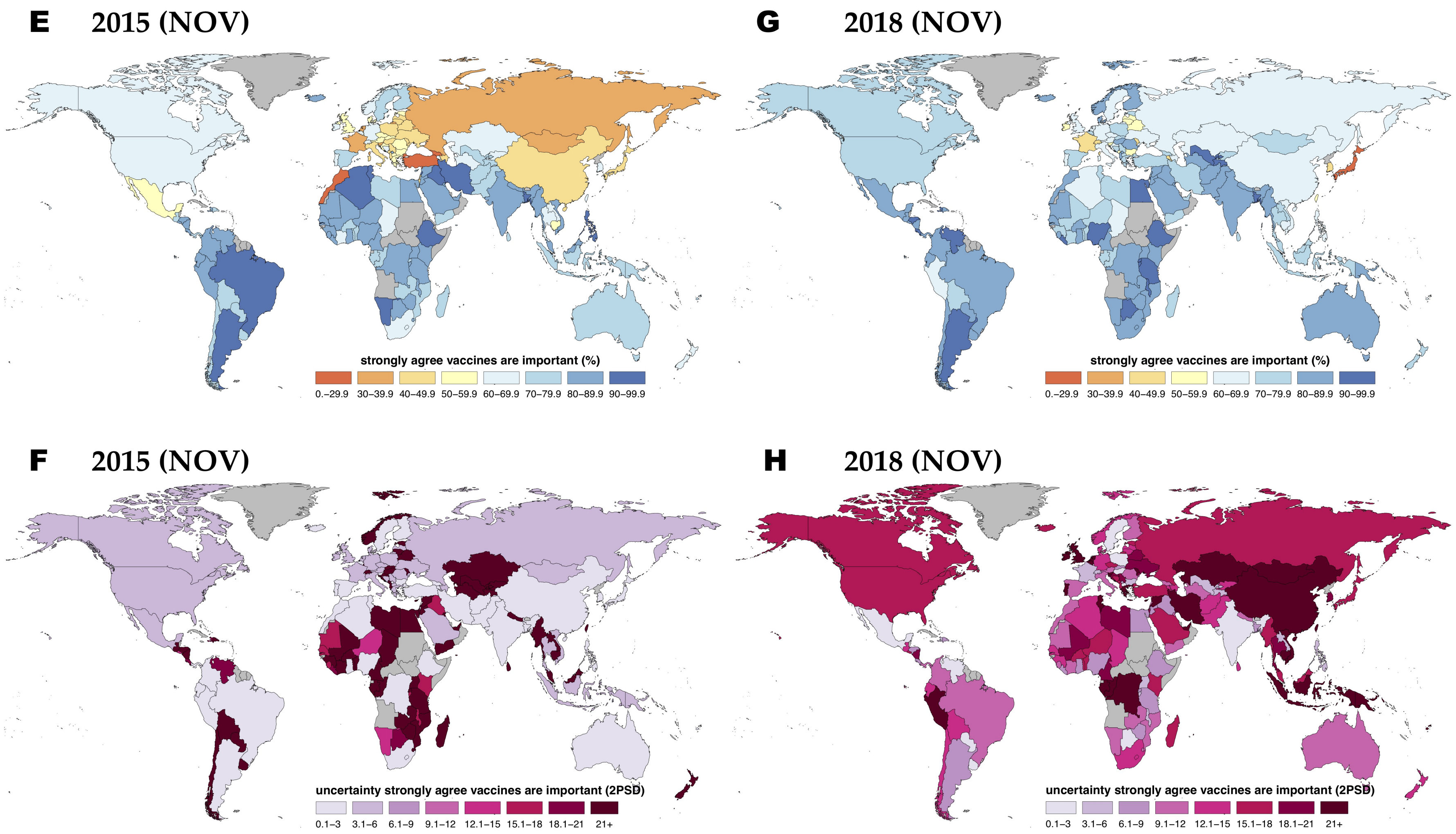

Figure 2: **E** Model-based estimates of the percentage of respondents strongly agreeing that vaccines are important for children to have in November 2015 **F** Uncertainty in the estimate of the percentage of respondents strongly agreeing that vaccines are important for children to have in November 2015 (two standard deviations of the posterior predictive distribution [2PSD]) **G** Model-based estimates of the percentage of respondents strongly agreeing that vaccines are important for children to have in November 2018 **H** Uncertainty in the estimate of the percentage of respondents strongly agreeing that vaccines are important for children to have in November 2019. No data were available for countries in grey.

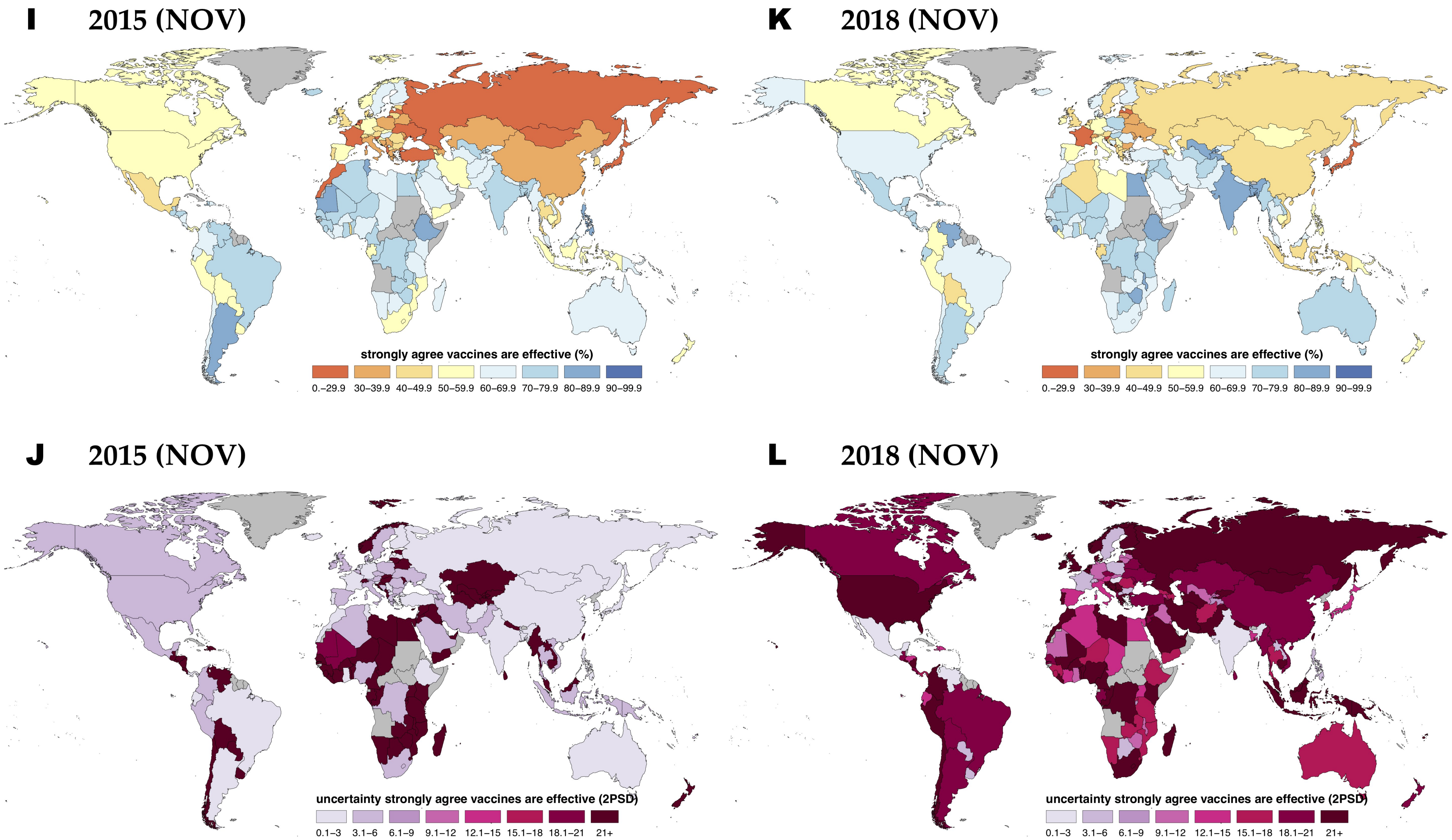

Figure 3: **I** Model-based estimates of the percentage of respondents strongly agreeing that vaccines are effective in November 2015 **J** Uncertainty in the estimate of the percentage of respondents strongly agreeing that vaccines are effective in November 2015 (two standard deviations of the posterior predictive distribution [2PSD]) **K** Model-based estimates of the percentage of respondents strongly agreeing that vaccines are effective in November 2018 **L** Uncertainty in the estimate of the percentage of respondents strongly agreeing that vaccines are effective in November 2019. No data were available for countries in grey.

4 Global trends in vaccine confidence

4.1 I think vaccines are safe

Figure 4 below (split over three pages), shows the model-based estimates and 95% highest posterior density (HPD) intervals for the statement *I think vaccines are safe.* Results are shown from all 149 countries from November 2015 to December 2019. This figure is an extended version of the maintext Figure 3A to show all countries across the world.

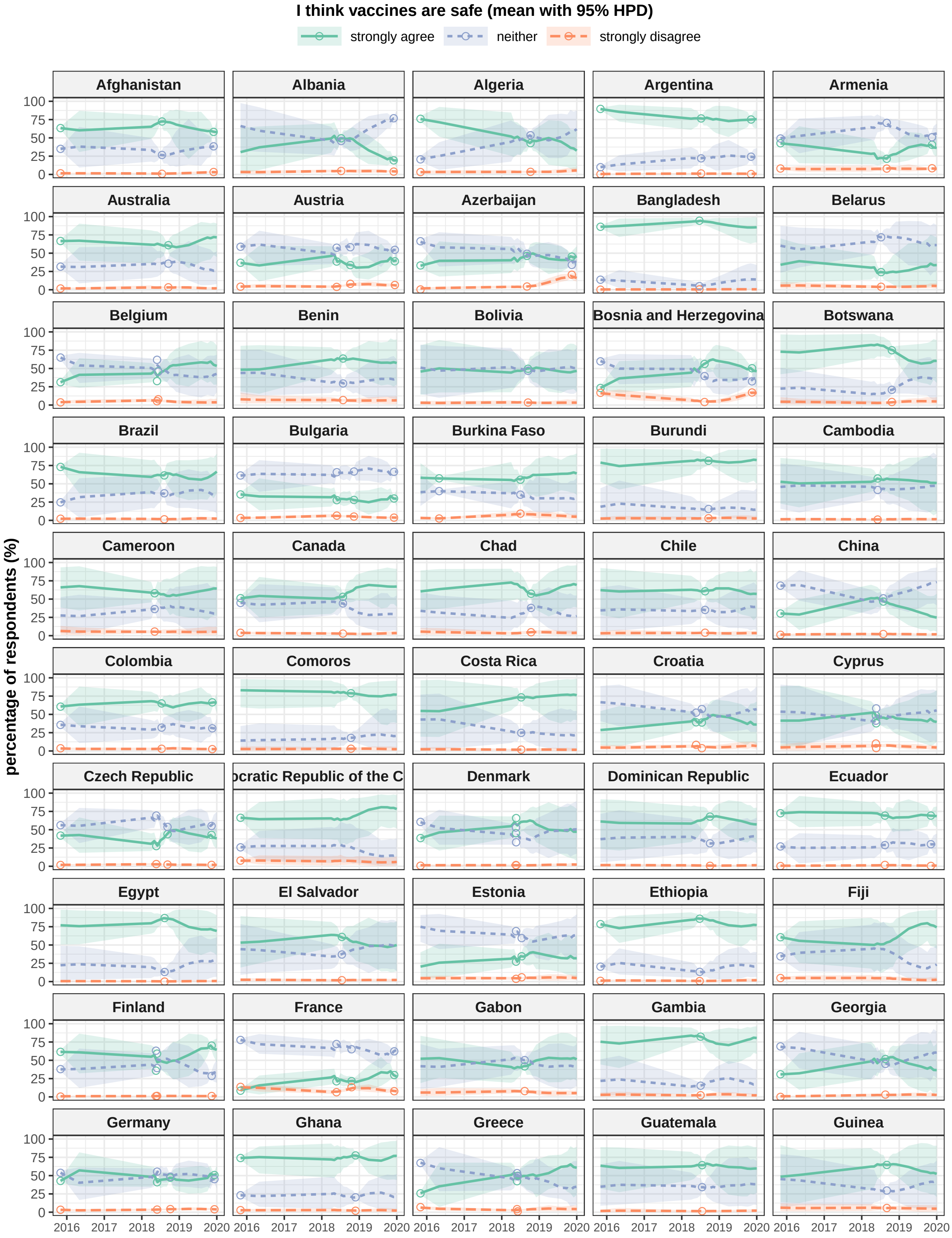
I think vaccines are safe (mean with 95% HPD)
strongly agree
neither
strongly disagree
percentage of respondents (%)
Afghanistan
Albania
Algeria
Argentina
Armenia
Australia
Austria
Azerbaijan
Bangladesh
Belarus
Belgium
Benin
Bolivia
Bosnia and Herzegovina
Botswana
Brazil
Bulgaria
Burkina Faso
Burundi
Cambodia
Cameroon
Canada
Chad
Chile
China
Colombia
Comoros
Costa Rica
Croatia
Cyprus
Czech Republic
ocratic Republic of the C
Denmark
Dominican Republic
Ecuador
Egypt
El Salvador
Estonia
Ethiopia
Fiji
Finland
France
Gabon
Gambia
Georgia
Germany
Ghana
Greece
Guatemala
Guinea
0
25
50
75
100
2016
2017
2018
2019
2020

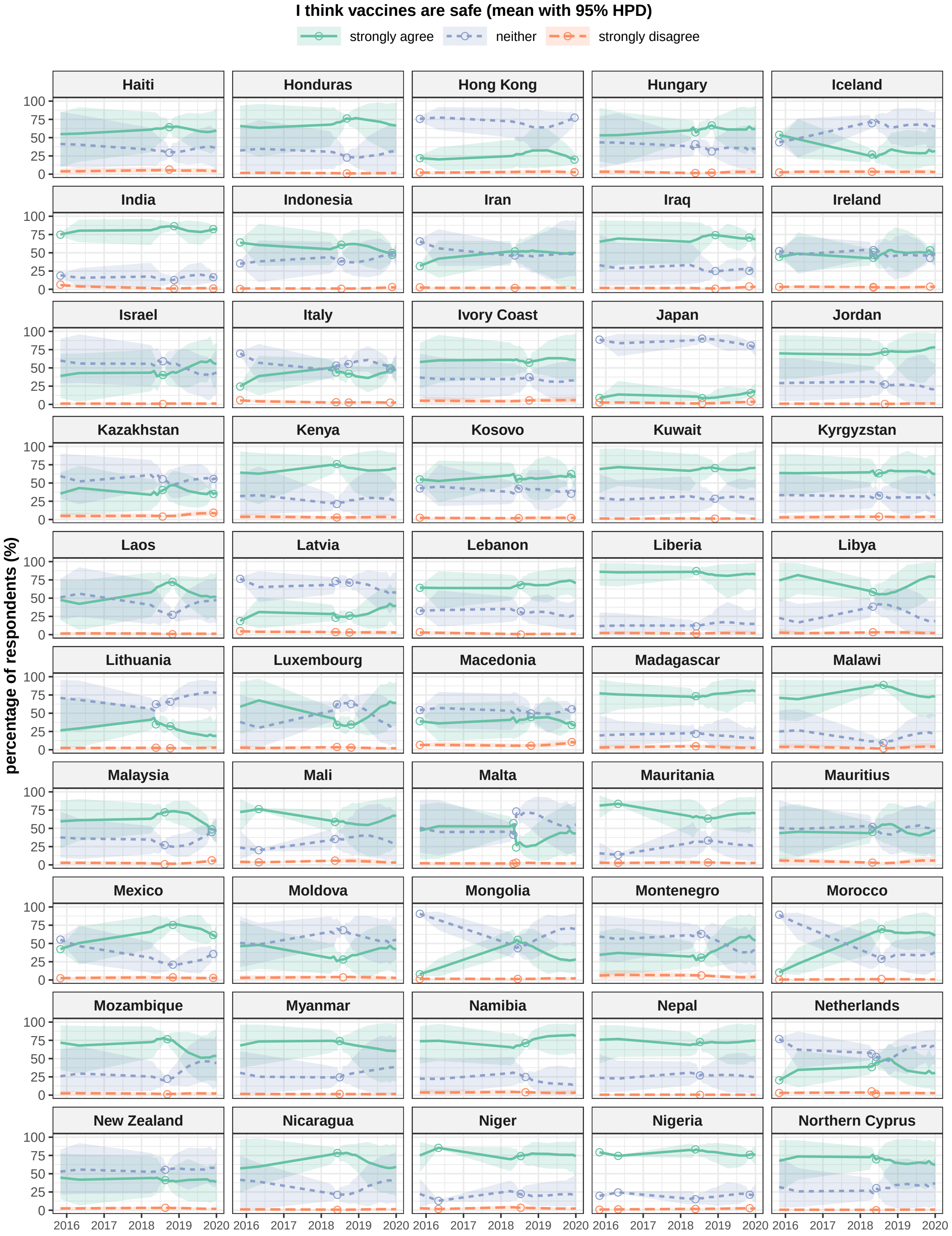
I think vaccines are safe (mean with 95% HPD)
strongly agree
neither
strongly disagree
percentage of respondents (%)
Haiti
Honduras
Hong Kong
Hungary
Iceland
India
Indonesia
Iran
Iraq
Ireland
Israel
Italy
Ivory Coast
Japan
Jordan
Kazakhstan
Kenya
Kosovo
Kuwait
Kyrgyzstan
Laos
Latvia
Lebanon
Liberia
Libya
Lithuania
Luxembourg
Macedonia
Madagascar
Malawi
Malaysia
Mali
Malta
Mauritania
Mauritius
Mexico
Moldova
Mongolia
Montenegro
Morocco
Mozambique
Myanmar
Namibia
Nepal
Netherlands
New Zealand
Nicaragua
Niger
Nigeria
Northern Cyprus
0
25
50
75
100
2016
2017
2018
2019
2020

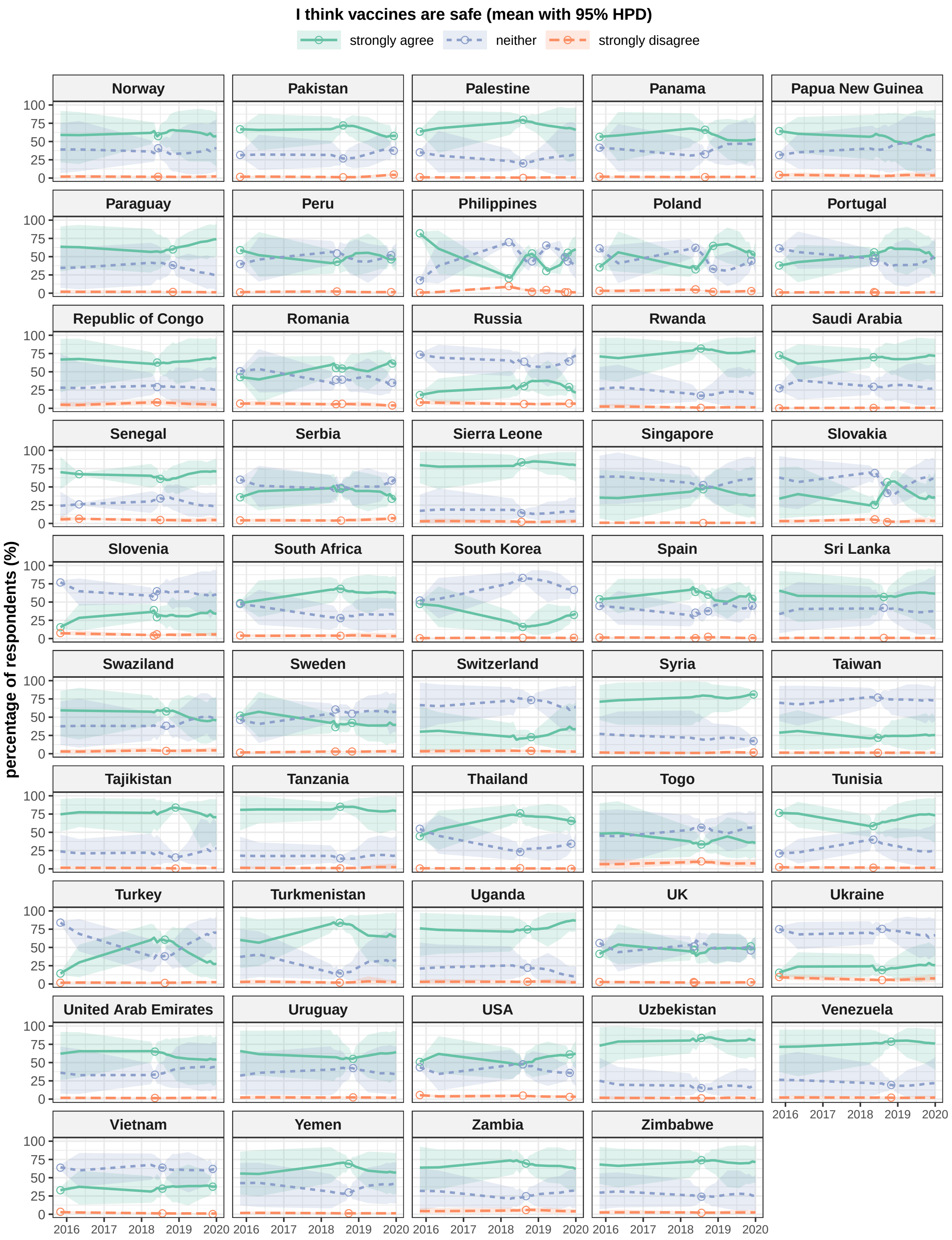

Figure 4: Time-series of estimated percentages of respondents strongly agreeing (green; solid line), strongly disagreeing (red; dashed), or neither strongly agreeing nor strongly disagreeing (blue; dotted) that vaccines are safe. The mean (line) and 95% HPD of the posterior predictive intervals (shaded regions) are shown. Circles show the observed percentage of respondents from raw data (see SM data).

4.2 I think vaccines are important

Figure 5 below (again split over three pages), shows the model-based estimates and 95% highest posterior density (HPD) intervals for the statement *I think vaccines are important for children to have.* Results are shown from all 149 countries from November 2015 to December 2019. This figure is an extended version of the maintext Figure 3A to show all countries across the world.

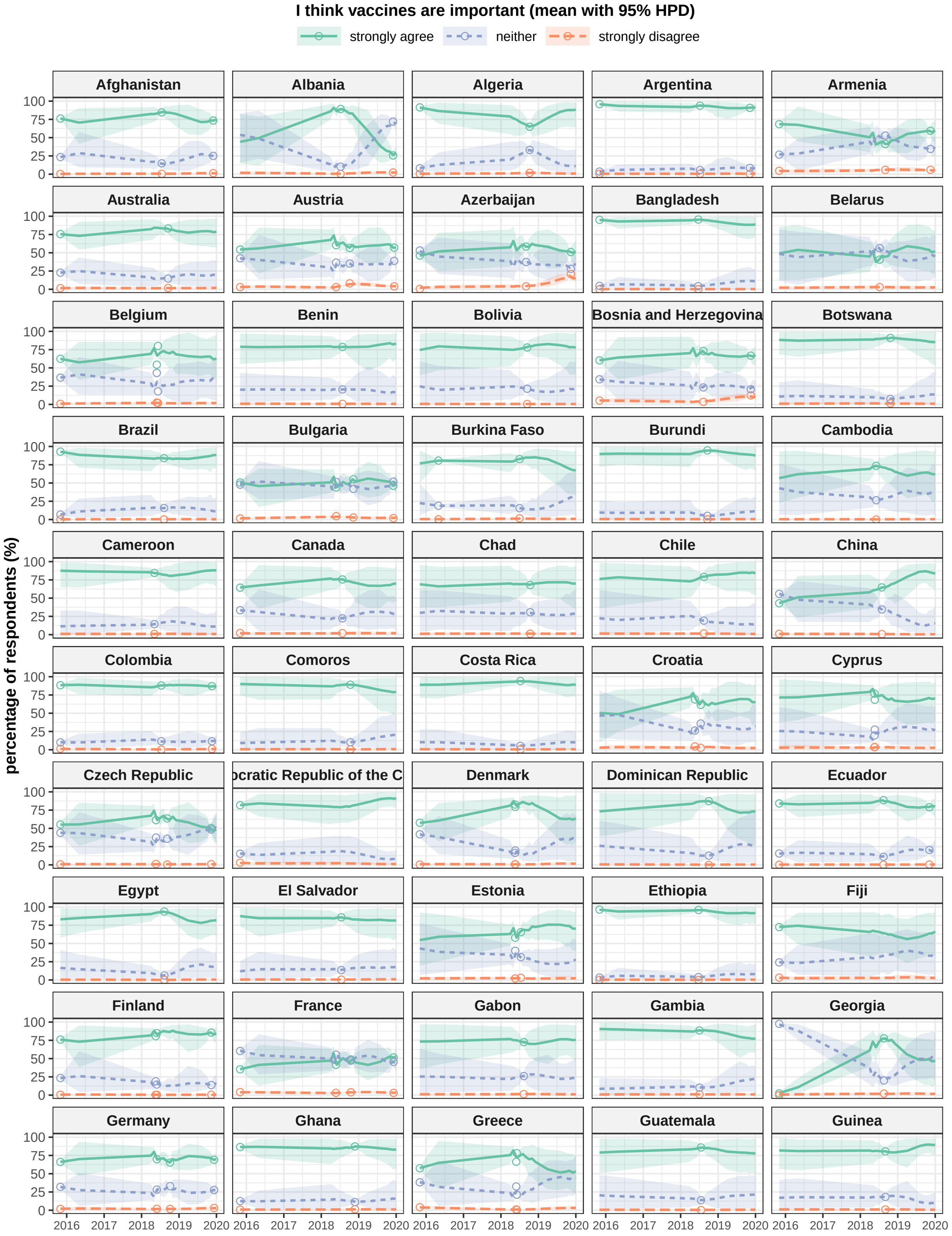
I think vaccines are important (mean with 95% HPD)
strongly agree
neither
strongly disagree
percentage of respondents (%)
Afghanistan
Albania
Algeria
Argentina
Armenia
Australia
Austria
Azerbaijan
Bangladesh
Belarus
Belgium
Benin
Bolivia
Bosnia and Herzegovina
Botswana
Brazil
Bulgaria
Burkina Faso
Burundi
Cambodia
Cameroon
Canada
Chad
Chile
China
Colombia
Comoros
Costa Rica
Croatia
Cyprus
Czech Republic
ocratic Republic of the C
Denmark
Dominican Republic
Ecuador
Egypt
El Salvador
Estonia
Ethiopia
Fiji
Finland
France
Gabon
Gambia
Georgia
Germany
Ghana
Greece
Guatemala
Guinea
0
25
50
75
100
2016
2017
2018
2019
2020

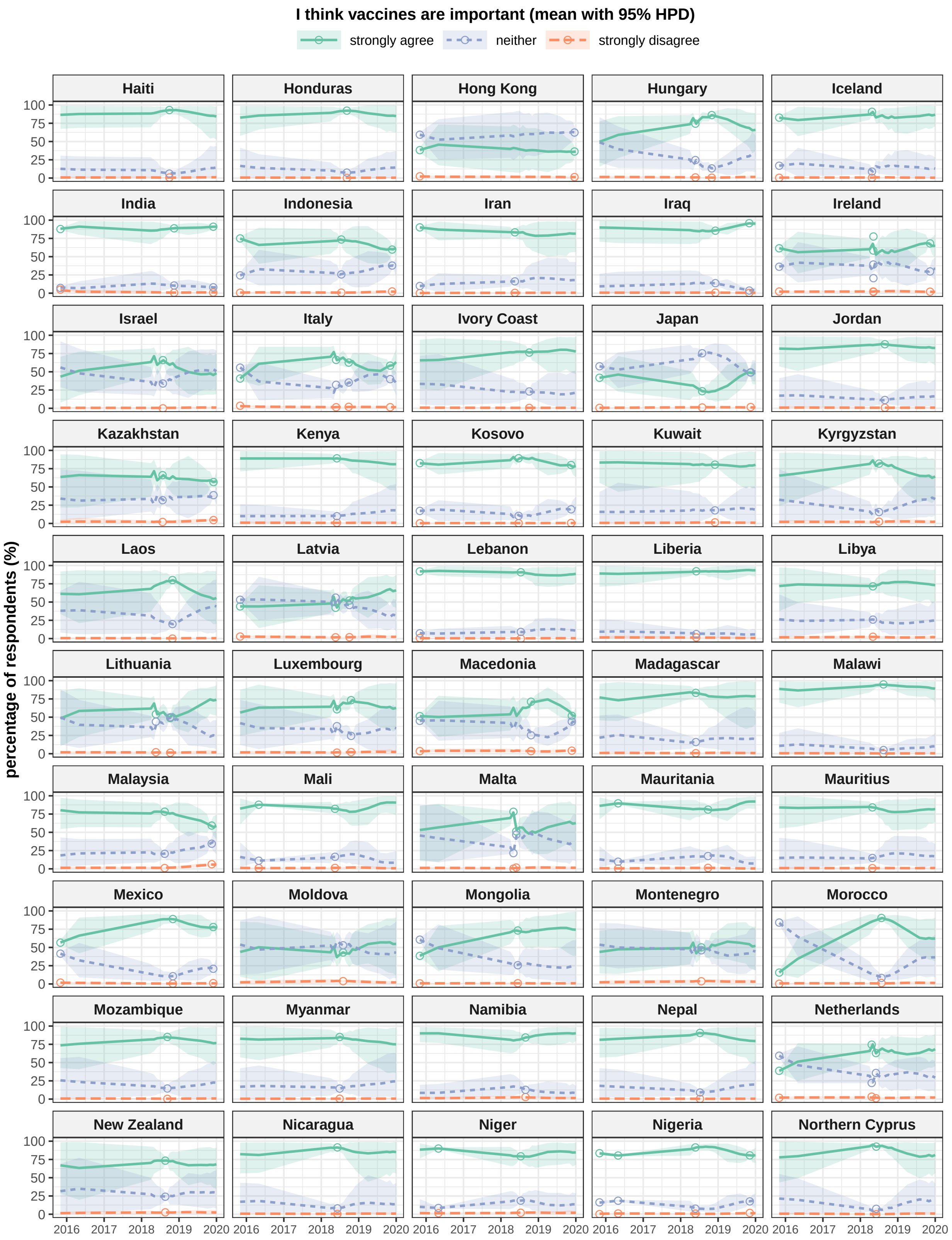
I think vaccines are important (mean with 95% HPD)
strongly agree
neither
strongly disagree
percentage of respondents (%)
Haiti
Honduras
Hong Kong
Hungary
Iceland
India
Indonesia
Iran
Iraq
Ireland
Israel
Italy
Ivory Coast
Japan
Jordan
Kazakhstan
Kenya
Kosovo
Kuwait
Kyrgyzstan
Laos
Latvia
Lebanon
Liberia
Libya
Lithuania
Luxembourg
Macedonia
Madagascar
Malawi
Malaysia
Mali
Malta
Mauritania
Mauritius
Mexico
Moldova
Mongolia
Montenegro
Morocco
Mozambique
Myanmar
Namibia
Nepal
Netherlands
New Zealand
Nicaragua
Niger
Nigeria
Northern Cyprus
0
25
50
75
100
2016
2017
2018
2019
2020

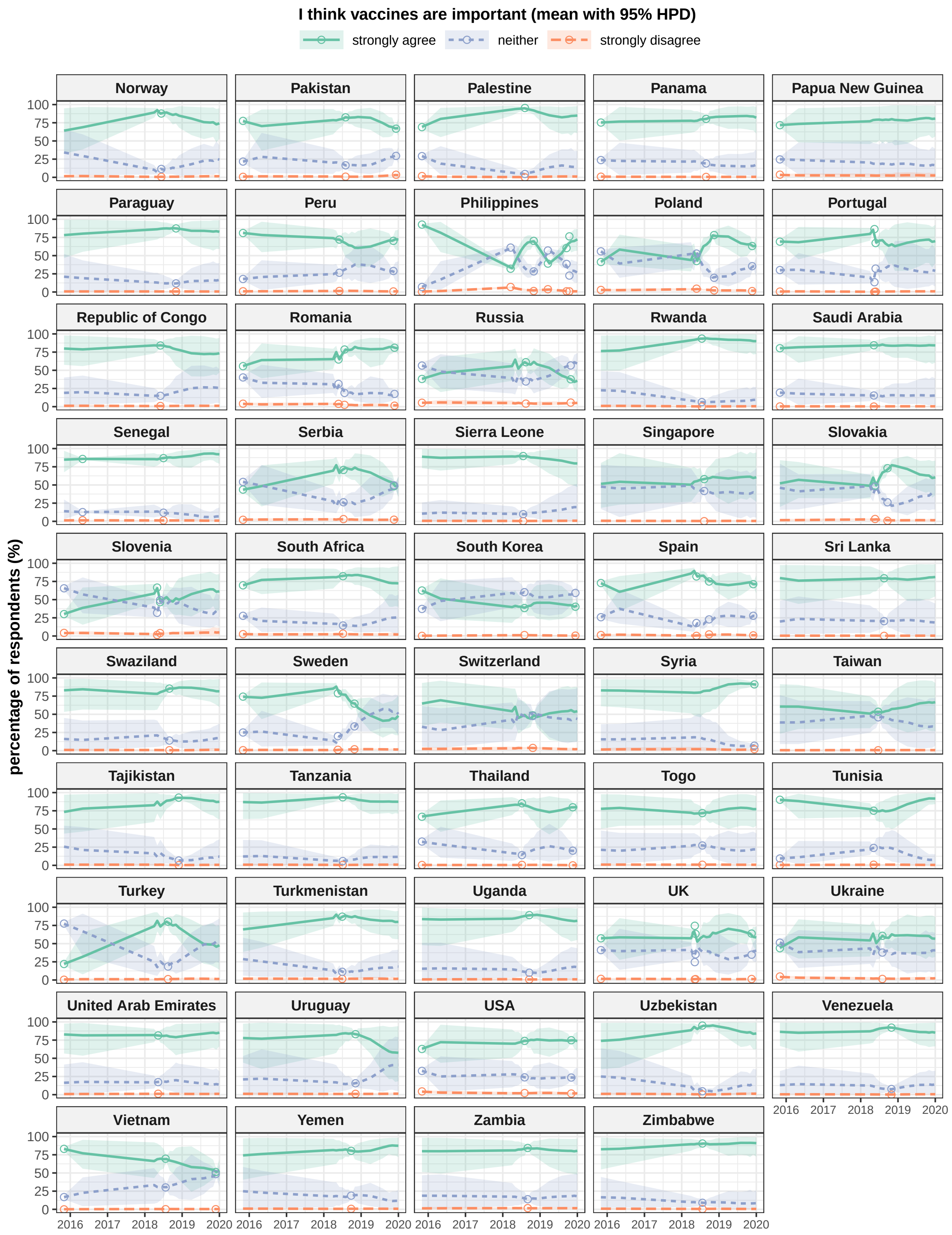

Figure 5: Time-series of estimated percentages of respondents strongly agreeing (green; solid line), strongly disagreeing (red; dashed), or neither strongly agreeing nor strongly disagreeing (blue; dotted) that vaccines are important for children to have. The mean (line) and 95% HPD of the posterior predictive intervals (shaded regions) are shown. Circles show the observed percentage of respondents from raw data (see SM data).

4.3 I think vaccines are effective

Figure 6 below (again split over three pages), shows the model-based estimates and 95% highest posterior density (HPD) intervals for the statement *I think vaccines are effective.* Results are shown from all 149 countries from November 2015 to December 2019. This figure is an extended version of the maintext Figure 3A to show all countries around the world.

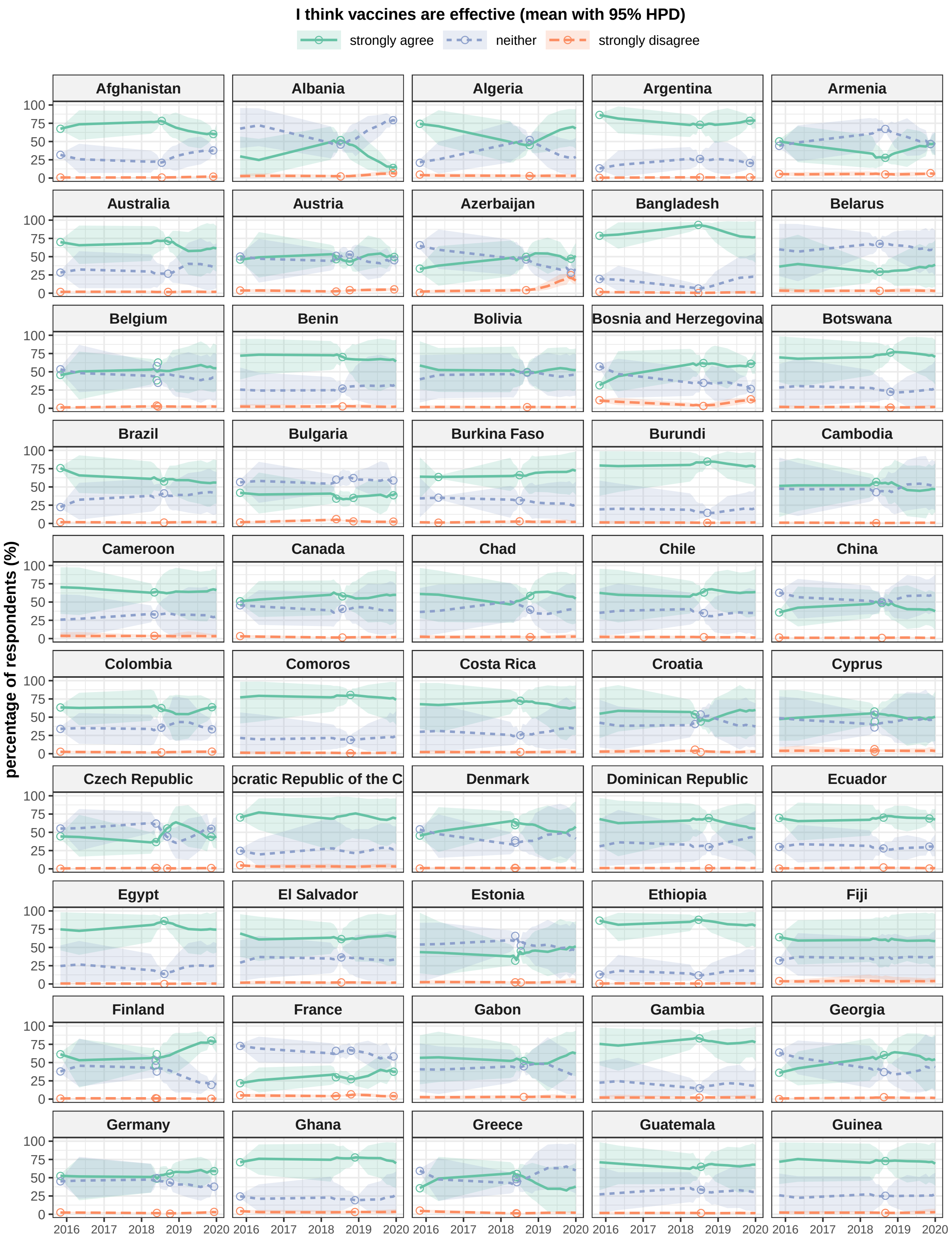

I think vaccines are effective (mean with 95% HPD)
strongly agree
neither
strongly disagree
percentage of respondents (%)
Afghanistan
Albania
Algeria
Argentina
Armenia
Australia
Austria
Azerbaijan
Bangladesh
Belarus
Belgium
Benin
Bolivia
Bosnia and Herzegovina
Botswana
Brazil
Bulgaria
Burkina Faso
Burundi
Cambodia
Cameroon
Canada
Chad
Chile
China
Colombia
Comoros
Costa Rica
Croatia
Cyprus
Czech Republic
ocratic Republic of the C
Denmark
Dominican Republic
Ecuador
Egypt
El Salvador
Estonia
Ethiopia
Fiji
Finland
France
Gabon
Gambia
Georgia
Germany
Ghana
Greece
Guatemala
Guinea
0
25
50
75
100
2016
2017
2018
2019
2020

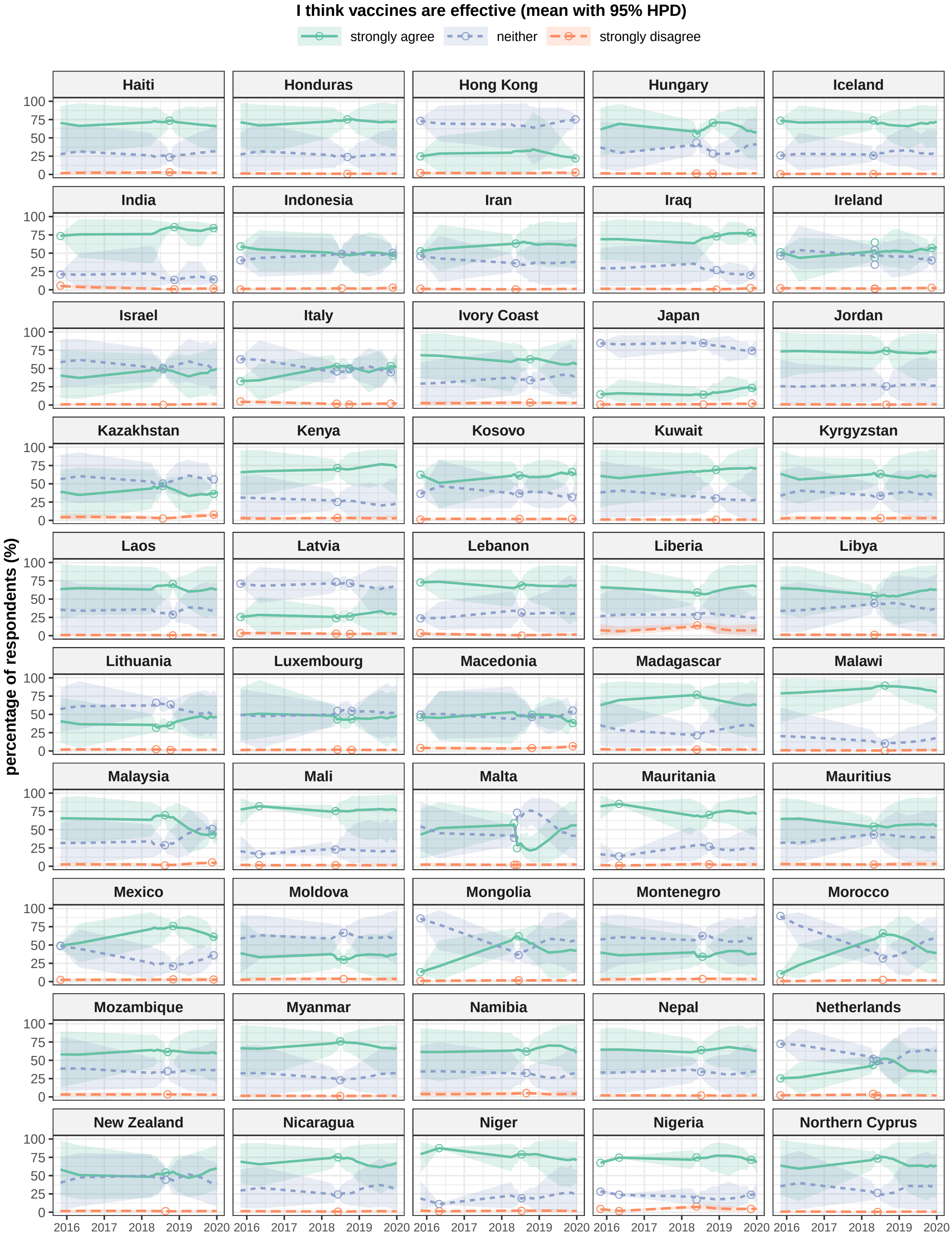
I think vaccines are effective (mean with 95% HPD)
strongly agree
neither
strongly disagree
percentage of respondents (%)
Haiti
Honduras
Hong Kong
Hungary
Iceland
India
Indonesia
Iran
Iraq
Ireland
Israel
Italy
Ivory Coast
Japan
Jordan
Kazakhstan
Kenya
Kosovo
Kuwait
Kyrgyzstan
Laos
Latvia
Lebanon
Liberia
Libya
Lithuania
Luxembourg
Macedonia
Madagascar
Malawi
Malaysia
Mali
Malta
Mauritania
Mauritius
Mexico
Moldova
Mongolia
Montenegro
Morocco
Mozambique
Myanmar
Namibia
Nepal
Netherlands
New Zealand
Nicaragua
Niger
Nigeria
Northern Cyprus
0
25
50
75
100
2016
2017
2018
2019
2020

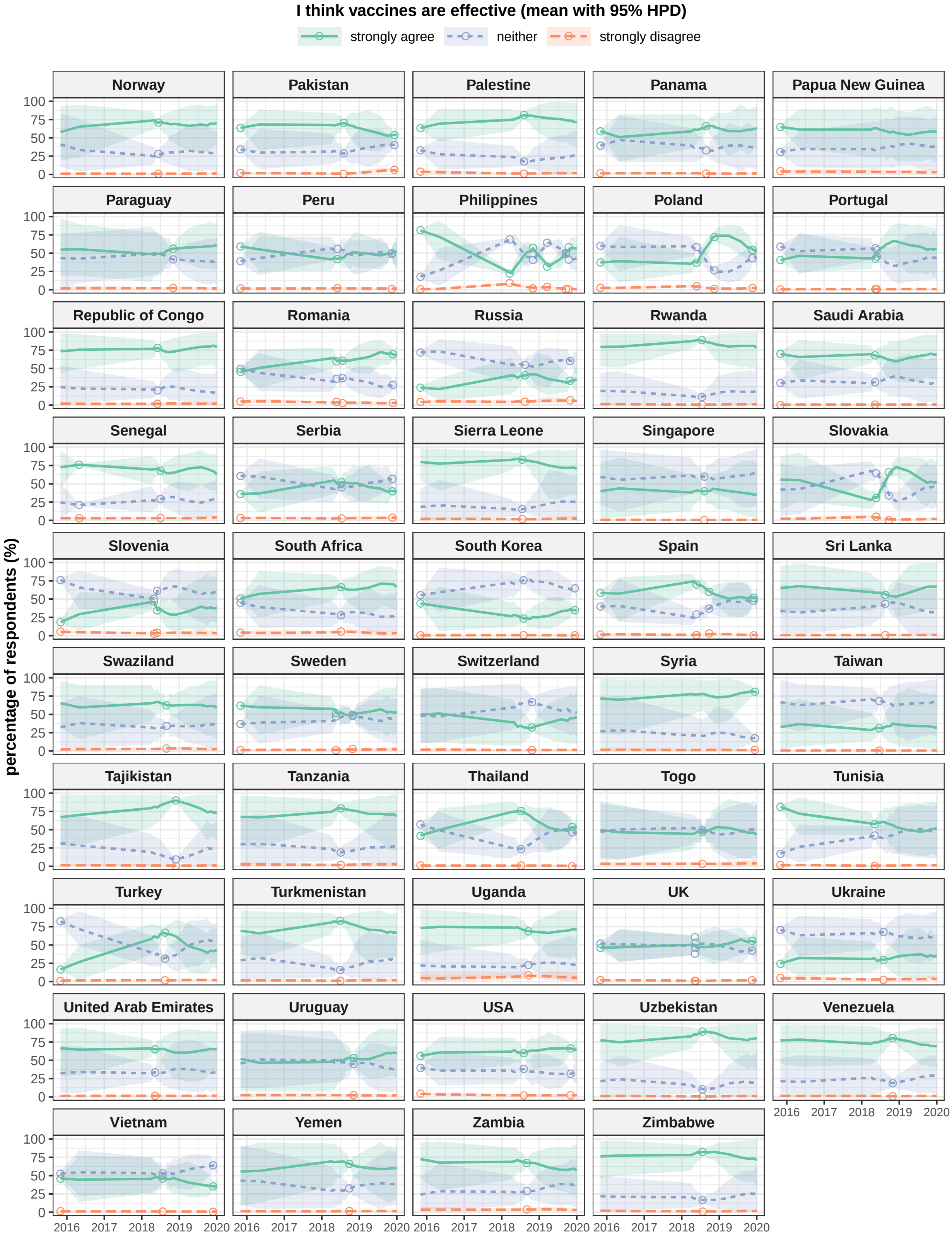

Figure 6: Time-series of estimated percentages of respondents strongly agreeing (green; solid line), strongly disagreeing (red; dashed), or neither strongly agreeing nor strongly disagreeing (blue; dotted) that vaccines are effective. The mean (line) and 95% HPD of the posterior predictive intervals (shaded regions) are shown. Circles show the observed percentage of respondents from raw data (see SM data).

4.4 Decreases in vaccine confidence

Our model identifies 10 countries (Afghanistan, Azerbaijan, Bosnia and Herzegovina, Georgia, Indonesia, Japan, Malaysia, Nigeria, Pakistan, Serbia) where there is a significant[3] increase in the percentage of respondents strongly disagreeing that vaccines are either safe, effective, or important in December, 2019, compared with four years earlier (see Figures 7, 8, and 9 below).

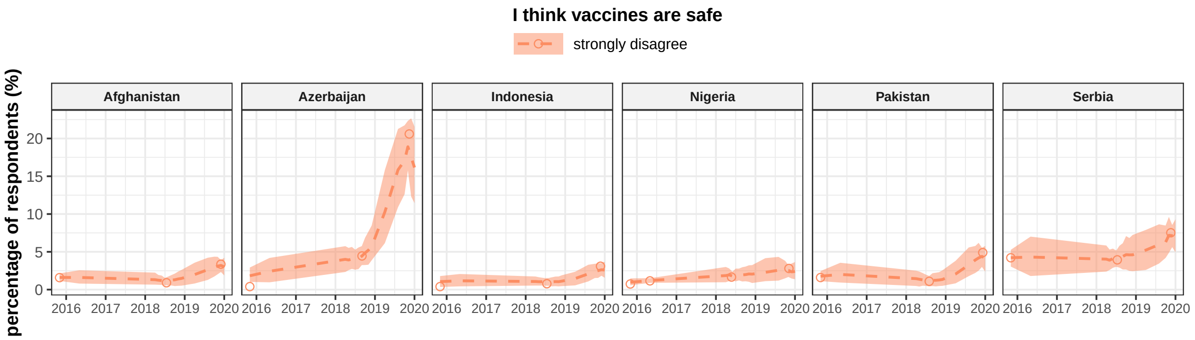

Figure 7: **Countries with significant increases in the proportion of respondents strongly disagreeing that vaccines are safe between November 2015 and December 2019.**

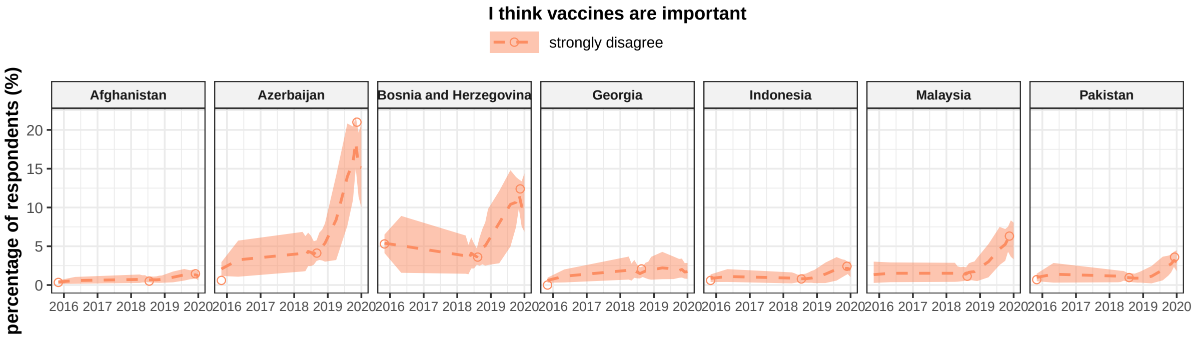

Figure 8: **Countries with significant increases in the proportion of respondents strongly disagreeing that vaccines are important between November 2015 and December 2019.**

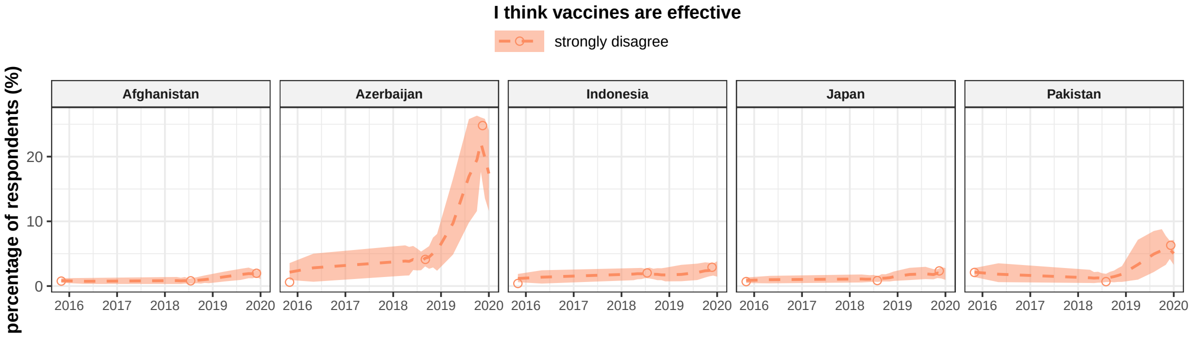

Figure 9: **Countries with significant increases in the proportion of respondents strongly disagreeing that vaccines are effective between November 2015 and December 2019.**

[3] A change is deemed significant if 95% of the bootstrapped distribution in the differences between posterior distributions of strongly disagreeing that vaccines are safe (or important, or effective) over two time points excludes zero.

5 Vaccine uptake and its socioeconomic determinants

5.1 Data summaries

Across the Wellcome Global Monitor dataset, all respondents who report having heard of vaccinations and having had children are considered for the univariate regression analysis between vaccine uptake and its determinants (maintext, Table 1). Across all countries, and among all individuals who reported ever having heard of vaccinations, a total of 92,656 individuals reported having children

A summary of these data across all countries are provided in the supplementary data file and a missing data summary is given below

5.2 Missing data

Across all countries the missing data fractions are low, with no more than 0.2% missing data for any variable (and often an order of magnitude lower). These missing fractions do vary by country; however there are only a small number of countries for which there is more than 1% missing data for any explanatory variable. These are listed below. As the missing data fractions are so low (with a few notable exceptions), we opt for a complete-case (deleting all observations with at least one missing value) analysis (See Methods in the main script).

Age

1-2%: Tanzania, Namibia, Zambia, Ireland, Mauritania, Burkina Faso, Comoros, The Gambia, Mali, Cameroon

2-4%: United Arab Emirates, Benin, Mozambique, Congo Brazzaville, Guinea, Gabon, Kuwait

4+%: Niger (4.3%), Lithuania (8.1%)

Income

All missing: Venezuela, Mauritania, Niger

Religion

4+%: Saudi Arabia (6.6%), Kuwait (13.3%), UAE (93.1%)

Education

1-2%: Iceland, Congo Brazzaville, Cameroon, Algeria, Burundi, Guinea, Norway

2-4%: Mauritania, Gabon

4+%: Lithuania (4.2%), Niger (4.5%)

Trust traditional healers

1-2%: Guatemala, Israel, Latvia, Mauritania, Malawi

Trust government

1-2%: Greece, Montenegro, Mauritania, Belarus

2-4%: Latvia

All missing: Saudi Arabia

Trust HCWs

1-2%: Mauritania

Information-seeking behaviours

1-2%: Pakistan, Tajikistan

5.3 A note on Bayesian logistic regression

Bayesian logistic regression is used to investigate the link between uptake (a binary variable) and confidence in vaccines (confidence); sources of trust in, for example, healthcare workers and traditional healers (sources of trust); information-seeking behaviours (information-seeking); and socio-economic information (socio-economics). (See maintext Table 1 for full details). All regression parameters are discrete with the exception of age (which are integer-valued ages). Age is scaled to have mean zero and unit standard deviation. Normal $\mathcal{N}(0,1)$ priors are used for each regression parameter. Posterior distributions over these parameters are obtained via Gibbs sampling using the language JAGS [13]. In all models 5,000 burn in steps and 50,000 sampling iterations are used after successful adaptation. Convergence and burn in are verified using the Geweke statistic.

6 Vaccines in Wellcome Global Monitor

Albania (polio, measles, or tuberculosis), **Angola** (measles, rubella, or polio), **Argentina** (tuberculosis, polio, or measles), **Armenia** (hepatitis B, tuberculosis, or poliomyelitis), **Australia** (measles, mumps, or rubella), **Austria** (the flu, tetanus, or measles), **Azerbaijan** (polio, measles, or hepatitis), **Belarus** (hepatitis B, tuberculosis, or tetanus), **Belgium** (influenza, polio, or measles), **Benin** (tetanus, measles, or polio), **Bolivia** (yellow fever (fiebre amarilla), measles (sarampión), or influenza A / AH1N1), **Bosnia and Herzegovina** (polio, tuberculosis, or whooping cough), **Botswana** (tuberculosis, measles, or polio), **Brazil** (tuberculosis, poliomyelitis, or MMR), **Bulgaria** (hepatitis B, tuberculosis, or polio), **Burkina Faso** (measles, tetanus, or polio), **Burundi** (measles, tetanus, or polio), **Cambodia** (tuberculosis, measles, or polio), **Cameroon** (measles, polio, or tuberculosis), **Canada** (tetanus, diphtheria, or whooping cough), **Chad** (tetanus, measles, or polio), **Chile** (measles, influenza, or hepatitis), **China** (hepatitis B, tuberculosis, or polio), **Colombia** (hepatitis, tetanus, or measles), **Comoros** (measles, polio, or meningitis), **Congo-Brazzaville** (measles, tuberculosis, or polio), **Costa Rica** (influenza, measles, or tetanus), **Croatia** (measles, mumps, or tuberculosis), **Cyprus** (tetanus, measles, or mumps), **Czech Republic** (tuberculosis, hepatitis, or meningococcus), **Denmark** (polio, meningitis, or measles), **Dominican Republic** (pentavalent, DPT, or measles), **Ecuador** (chickenpox, measles, or influenza/rotavirus), **El Salvador** (hepatitis B, neumococo infantil, or polio), **Estonia** (polio, measles, or mumps), **Ethiopia** (tuberculosis, polio, or measles), **Finland** (polio, measles, or mumps), **France** (diphtheria, tetanus, or poliomyelitis), **Gabon** (measles, tuberculosis, or polio), **Gambia** (measles, tetanus, or polio), **Georgia** (polio, hepatitis B, or measles), **Germany** (flu, tetanus, or measles), **Ghana** (polio, measles, or meningitis), **Greece** (poliomyelitis, measles, or rubella), **Guatemala** (tuberculosis (BCG), hepatitis B, or poliomyelitis), **Guinea** (measles, tetanus, or polio), **Haiti** (measles, pneumonia, or poliomyelitis), **Honduras** (neumococo, rotavirus, or pentavalente), **Hungary** (tuberculosis, pertussis, or measles), **Iceland** (tetanus, polio, or pertussis), **Indonesia** (tuberculosis, hepatitis, or tetanus), **Ireland** (diphtheria, tetanus, or polio), **Italy** (poliomyelitis, tetanus, or measles), **Ivory Coast** (rubella, measles, or rotavirus), **Japan** (tetanus, polio, or encephalitis B), **Kazakhstan** (polio, measles, or mumps), **Kenya** (tuberculosis, polio, or measles), **Kosovo** (tuberculosis, hepatitis, or polio), **Kyrgyzstan** (polio, measles, or mumps), **Laos** (malaria, pneumonia, or influenza), **Latvia** (chicken pox, measles, or mumps), **Liberia** (measles, hepatitis B, or tuberculosis), **Lithuania** (polio, roseola, or measles), **Luxembourg** (flu, tetanus, or measles), **Macedonia** (tuberculosis, hepatitis B, or polio), **Madagascar** (diphtheria, pneumonia, or rubella), **Malawi** (polio, measles, or tuberculosis), **Mali** (measles, polio, or tuberculosis), **Malta** (MMR, tetanus, or influenza), **Mauritania** (measles, tetanus, or polio), **Mauritius** (mumps, rubella, or pertussis), **Mexico** (polio, tetanus or measles), **Moldova** (measles, polio, or pneumonia), **Mongolia** (polio, measles, or diphtheria), **Montenegro** (polio, measles, or mumps), **Mozambique** (tetanus, measles, or polio), **Namibia** (diphtheria, hepatitis B, or polio), **Nepal** (polio, measles, or tuberculosis), **Netherlands** (polio, measles, or mumps), **New Zealand** (measles, mumps, or rubella), **Nicaragua** (polio, malaria, or rubella), **Niger** (tetanus, polio, or meningitis), **Nigeria** (polio, measles, or meningitis), **Northern**

Cyprus (chickenpox, epidemic parotitis, or measles), **Norway** (diphtheria, meningitis, or measles), **Panama** (poliomyelitis, hepatitis, pneumococcus), **Paraguay** (polio, varicella, or hepatitis), **Peru** (hepatitis, flu, or polio), **Philippines** (polio, measles, or tuberculosis), **Poland** (hepatitis B, tuberculosis, or measles), **Portugal** (tuberculosis, hepatitis B, or polio), **Romania** (tuberculosis, tetanus, or diphtheria), **Russia** (chickenpox, measles, or rubella), **Rwanda** (measles, tetanus, or meningitis), **Senegal** (measles, tetanus, or polio), **Serbia** (measles, tuberculosis, or polio), **Sierra Leone** (diphtheria, measles, or polio), **Singapore** (influenza, tuberculosis, or hepatitis), **Slovakia** (measles, mumps, or rubella), **Slovenia** (measles, tetanus, or chickenpox), **South Africa** (tuberculosis, measles, or polio), **South Korea** (chicken pox, hepatitis, or pneumonia), **Spain** (polio, measles, or tetanus), **Swaziland** (tuberculosis, measles, or polio), **Sweden** (diphtheria, measles, or tetanus), **Switzerland** (flu, tetanus, or measles), **Taiwan** (measles, hepatitis B, or polio), **Tajikistan** (poliomyelitis, tetanus, or diphtheria), **Tanzania** (tuberculosis, measles, or hepatitis), **Thailand** (tetanus, dengue fever, or polio), **Togo** (rubella, measles, or tetanus), **Turkmenistan** (polio, measles, or mumps), **UK** (diphtheria, polio, or tetanus), **Uganda** (tuberculosis, polio, or measles), **Ukraine** (poliomyelitis, measles, or rubella), **United States** (polio, influenza, or Hepatitis B), **Uruguay** (measles, rubella, or mumps), **Uzbekistan** (hepatitis B, tetanus, or tuberculosis), **Venezuela** (hepatitis A-B, tuberculosis, or polio), **Vietnam** (hepatitis B, measles, or polio), **Zambia** (polio, measles, or cholera), **Zimbabwe** (diphtheria, measles, or polio)

References

[1] http://www.gallup-international.com/surveys/global-leaders/ [accessed 11 November 2019], "39th end of year survey," 2015.

[2] H. J. Larson, A. De Figueiredo, Z. Xiahong, W. S. Schulz, P. Verger, I. G. Johnston, A. R. Cook, and N. S. Jones, "The state of vaccine confidence 2016: global insights through a 67-country survey," *EBioMedicine*, vol. 12, pp. 295–301, 2016.

[3] H. Larson, A. de Figueiredo, E. Karafillakis, and M. Rawal, "State of vaccine confidence in the eu 2018," *Luxembourg: Publications Office of the European Union https://ec. europa. eu/health/sites/health/files/vaccination/docs/2018_vaccine_confidence_en. pdf*, 2018.

[4] https://wellcome.ac.uk/reports/wellcome-global-monitor/2018 [accessed 11 November 2019], "Wellcome global monitor, 2018.," 2018.

[5] https://wellcome.ac.uk/reports/wellcome-global-monitor/2018 [accessed 11 November 2019], "Wellcome global monitor, appendices 2018. appendix a methodology.," 2018.

[6] https://www.psrc.com.ph [accessed 11 November 2019], "Philippines survey and research center," 2019.

[7] "43rd end of year survey," 2019.

[8] C. K. Williams and D. Barber, "Bayesian classification with gaussian processes," *IEEE Transactions on Pattern Analysis and Machine Intelligence*, vol. 20, no. 12, pp. 1342–1351, 1998.

[9] J. Riihimäki, P. Jylänki, and A. Vehtari, "Nested expectation propagation for gaussian process classification with a multinomial probit likelihood," *Journal of Machine Learning Research*, vol. 14, no. Jan, pp. 75–109, 2013.

[10] C. Cargnoni, P. Müller, and M. West, "Bayesian forecasting of multinomial time series through conditionally gaussian dynamic models," *Journal of the American Statistical Association*, vol. 92, no. 438, pp. 640–647, 1997.

[11] H. J. Larson, K. Hartigan-Go, and A. de Figueiredo, "Vaccine confidence plummets in the philippines following dengue vaccine scare: why it matters to pandemic preparedness," *Human vaccines & immunotherapeutics*, vol. 15, no. 3, pp. 625–627, 2019.

[12] J. F. Geweke and K. J. Singleton, "Interpreting the likelihood ratio statistic in factor models when sample size is small," *Journal of the American Statistical Association*, vol. 75, no. 369, pp. 133–137, 1980.

[13] M. Plummer *et al.*, "Jags: A program for analysis of bayesian graphical models using gibbs sampling," in *Proceedings of the 3rd international workshop on distributed statistical computing*, no. 125 in 124, p. 10, Vienna, Austria., 2003.
